# Ecologically valid virtual reality-based technologies for assessment and rehabilitation of acquired brain injury: a systematic review

**DOI:** 10.3389/fpsyg.2023.1233346

**Published:** 2023-08-29

**Authors:** Ana Lúcia Faria, Jorge Latorre, Mónica Silva Cameirão, Sergi Bermúdez i Badia, Roberto Llorens

**Affiliations:** ^1^Faculdade de Artes e Humanidades, Universidade da Madeira, Funchal, Portugal; ^2^NOVA Laboratory for Computer Science and Informatics, Lisbon, Portugal; ^3^Agência Regional para o Desenvolvimento da Investigação, Tecnologia e Inovação, Funchal, Portugal; ^4^Neurorehabilitation and Brain Research Group, Instituto de Investigación e Innovación en Bioingeniería, Universitat Politècnica de València, Valencia, Spain; ^5^NEURORHB, Servicio de Neurorrehabilitación de Hospitales Vithas, Valencia, Spain; ^6^Faculdade de Ciências Exatas e da Engenharia, Universidade da Madeira, Funchal, Portugal

**Keywords:** ecological validity, virtual reality, assessment, rehabilitation, acquired brain injury, activities of daily living

## Abstract

**Purpose:**

A systematic review was conducted to examine the state of the literature regarding using ecologically valid virtual environments and related technologies to assess and rehabilitate people with Acquired Brain Injury (ABI).

**Materials and methods:**

A literature search was performed following the PRISMA guidelines using PubMed, Web of Science, ACM and IEEE databases. The focus was on assessment and intervention studies using ecologically valid virtual environments (VE). All studies were included if they involved individuals with ABI and simulated environments of the real world or Activities of Daily Living (ADL).

**Results:**

Seventy out of 363 studies were included in this review and grouped and analyzed according to the nature of its simulation, prefacing a total of 12 kitchens, 11 supermarkets, 10 shopping malls, 16 streets, 11 cities, and 10 other everyday life scenarios. These VE were mostly presented on computer screens, HMD’s and laptops and patients interacted with them primarily via mouse, keyboard, and joystick. Twenty-five out of 70 studies had a non-experimental design.

**Conclusion:**

Evidence about the clinical impact of ecologically valid VE is still modest, and further research with more extensive samples is needed. It is important to standardize neuropsychological and motor outcome measures to strengthen conclusions between studies.

**Systematic review registration:**

identifier CRD42022301560, https://www.crd.york.ac.uk/prospero/display_record.php?RecordID=301560.

## 1. Introduction

Given the high prevalence of cognitive impairment, functional dependence and social isolation after acquired brain injury (ABI), namely traumatic brain injury (TBI) (Robba and Citerio, 2023) and stroke (Tsao et al., 2023), finding effective motor and cognitive assessment and rehabilitation solutions has been a primary goal for many research studies in the field of health technologies (Bernhardt et al., 2019). Performance of many daily activities, such as doing the groceries, implies getting to outdoor locations, such as supermarkets or shopping malls. Street crossing and driving are demanding tasks that require multiple and complex cognitive skills that are commonly impaired after ABI (Parsons, 2015). Although the goal of rehabilitation is to improve individuals’ independence in these activities, their practice in real environments can be dangerous because of intrinsic hazards such as traffic or pedestrians, and are extremely resource-intensive in terms of staff management and financial costs, which are scarce in most clinics (Bohil et al., 2011). These limitations have motivated the use of Virtual Reality (VR) to safely recreate different scenarios in the clinic (Rizzo et al., 2004).

Most ABI rehabilitation approaches rely on theoretically valid principles, however the exercises and activities, such as physiotherapy and occupational therapy, repetitive motor exercises, and paper-and-pencil cognitive exercises with static stimuli, are demotivating and lack ecological validity (Parsons, 2016). The issue of ecological validity started being discussed already in 1982 when Neisser argued that cognitive psychology experiments were conducted in artificial settings and employed measures with no counterparts in everyday life (Neisser, 1982). In opposition, Banaji and Crowder (1989) advocated that ecological approaches lack the internal validity and experimental control needed for scientific progress (Banaji and Crowder, 1989). In 1996, Franzen and Wilhelm conceptualized ecological validity as having two aspects; veridicality, in which the person’s performance on a construct-driven measure should predict some feature(s) of the person’s everyday life functioning, and verisimilitude, in which the requirements of a neuropsychological measure and the testing conditions should resemble requirements found in a person’s ADL’s (Franzen and Wilhelm, 1996). Since then, the search for a balance between everyday activities and laboratory control has a long history in clinical neuroscience (Parsons, 2015) and researchers have been using different definitions and interpretations of the term ecological validity (Holleman et al., 2020).

A promising approach to improve neuropsychological assessment and rehabilitation ecological validity is the use of immersive and non-immersive VR systems (Rizzo et al., 2004; Parsons, 2015; Kourtesis and MacPherson, 2023). VR combines the control and rigor of laboratory measures with a simulation that depicts real life situations in a balance between naturalistic observation and the need for control key variables (Bohil et al., 2011). Over the last years, VR-based methodologies have been developed to assess and improve cognitive (Aminov et al., 2018; Luca et al., 2018; Maggio et al., 2019) and motor (Laver et al., 2015, 2017) functions, via immersive (e.g., Head Mounted Displays (HMDs), Cave Automatic Virtual Environment (CAVEs)) and non-immersive (2D computer screens, tablets, mobile phones) technologies. Non-immersive VR requires the use of controllers, joysticks and keyboards, which can be challenging for individuals with no gaming or pc-using experience, namely older adults and clinical populations (Werner and Korczyn, 2012; Zygouris and Tsolaki, 2015; Parsons et al., 2018; Laver et al., 2017; Zaidi et al., 2018). On the other hand, immersive VR using naturalistic interactions seems to facilitate comparable performance between gamers and non-gamers (Zaidi et al., 2018).

Some critical elements, such as presence, time perception, plausibility and embodiment, collectively contribute to the ecological validity of VR-based assessment and rehabilitation programs. Presence comprehends two illusions, usually referred to as Place Illusion (illusion of being in the place depicted by the VE) and Plausibility (illusion that the virtual events are really happening). Embodiment refers to the feeling of “owning” an avatar or virtual body. This aspect is particularly significant for patients with motor impairments. An embodied experience enhances motor learning and fosters a stronger mind–body connection during rehabilitation sessions (Vourvopoulos et al., 2022). Presence together with the embodiment are the key illusions of VR (Slater et al., 2022). Another important element is that time perception in VR differs from the physical world, leading to potential alterations in the patient’s temporal experience. Understanding how time is perceived in VR is crucial for designing effective assessment and rehabilitation protocols and managing patient expectations (Mullen and Davidenko, 2021). The integration of these key elements potentially enhances patient motivation and engagement, which might result in better adherence and improved outcomes.

According to Slater (1999) if a VR system allows the individual to turn their head in any direction at all and still receive visual information only from within the VE then it is a more immersive system than one where the individual can only see the VE visual signals along one fixed direction (Slater, 1999). Accordingly, while in a non-immersive VR system the VE is displayed on a computer monitor and the interaction is limited to a mouse, joystick or keyboard, in an immersive VR system (typically HMDs and CAVEs) the user is surrounded by a 3D representation of the real world and can use their own body for a naturalistic interaction. This strong feeling of ‘being physically present’ in the VE allows one to respond in a realistic way to the virtual stimuli (Agrewal et al., 2020), eliciting the activation of brain mechanisms that underlies sensorimotor integration and cerebral networks that regulate attention (Vecchiato et al., 2015). Notwithstanding the technical and theoretical differences between immersive and non-immersive VR, both have pros and cons concerning the use of novel assessment and rehabilitation systems to improve and personalize treatments according to the patient’s needs.

One of the explanations for the growing interest in VR is its potential to incorporate motor and cognitive tasks within the simulation of ADL’s, and to provide safe and controlled environments for patients to rehabilitate ADL’s. As part of their limitations, VR technologies may cause cybersickness symptomatology such as nausea, dizziness, disorientation, and postural instability. However, recent reviews and meta-analyses suggest that the symptoms are experienced due to the inappropriate implementation of immersive VR hardware and software (Kourtesis et al., 2019; Saredakis et al., 2020). Another problem is that researchers and clinicians do not quantitatively assess cybersickness and it can affect cognitive performance (Nesbitt et al., 2017; Arafat et al., 2018; Mittelstaedt et al., 2019). Since ABI patients may be more susceptible to experience these symptoms (Spreij et al., 2020b), there should be extra caution in the development of VR based assessment and intervention tools. Collaboration between clinicians, researchers, and technology developers is essential to produce VR based tools that can address the assessment and treatment need of the ABI patients (Zanier et al., 2018).

In the last years a number of reviews analyzed the use of ecologically-valid environments in cognitive and motor assessment and/or rehabilitation of multiple sclerosis (Weber et al., 2019), addictive disorders (Segawa et al., 2020), hearing problems (Keidser et al., 2020) mental health (Bell et al., 2022), language (Peeters, 2019) and neglect (Azouvi, 2017). In the last years some reviews have provided overviews within this field. Romero-Ayuso et al. (2021) conducted a review to determine the available tests for the assessment of executive functions with ecological validity to predict individuals’ functioning (Romero-Ayuso et al., 2021). The authors analyzed 76 studies and identified 110 tools to assess instrumental activities of daily living, namely menu preparation and shopping. Since they have focused in the executive functions assessment, they found a predominance of tests based on the Multiple Errands Test paradigm (Romero-Ayuso et al., 2021). Corti et al. (2022) have performed a review about the VR assessment tools for ABI, described in scientific publications between 2010 and 2019. Through the analysis of 38 studies they have identified 16 different tools that assessed executive functions and prospective memory and other 15 that assessed visuo-spatial abilities. The authors found that about half of these tools delivered tasks that differ from everyday life activities, limiting the generalization of the assessment to real world performance. Although the authors recognize great potential of VR for ABI assessment, they recommend the improvement of existing tools or development of new ones with more ecological validity (Corti et al., 2022).

To the best of our knowledge, no review has analyzed the characteristics and clinical validation of ecologically valid daily life scenarios developed to both assess and/or rehabilitate acquired brain injury patients concerning the cognitive and motor domains together. As such, this review aims to examine the state of the literature regarding the use of ecologically valid virtual environments to assess and rehabilitate people with ABI. This review will focus on (1) what are the most common virtual environments used in acquired brain injury assessment and rehabilitation, (2) which technologies are used for presentation and interaction in these environments, (3) how are these virtual environments being clinically validated about their impact in ABI assessment and rehabilitation.

## 2. Methods

A systematic search of the existing literature was performed following the PRISMA guidelines using four digital databases: PubMed, Web of Science, IEEE and ACM. The search focused on assessment and intervention studies published in English, from 2000 to 2021, in peer-reviewed journals and conferences. The search targeted titles and abstracts using the following keywords and boolean logic: ‘virtual reality’ OR ‘virtual environment’ OR ‘immersive’ AND ‘stroke’ OR ‘traumatic brain injury’ OR ‘acquired brain injury’ AND ‘rehabilitation’ OR ‘assessment’ AND ‘simulated environments’ OR ‘activities daily living’ NOT ‘motor’ OR ‘mobility’ OR ‘limb’ OR ‘balance’ OR ‘gait’.

All types of articles (not reviews and editorials) were included if: (1) they involved individuals of any age with stroke or TBI; (2) simulated environments of the real world or ADL and; (3) had at least one outcome measure result related to the intervention clinical effects. All simulated environments were considered, including 360° videos and serious games. We have included non-immersive (e.g., computer screen and tablet), semi-immersive (e.g., wall projections and driving simulators), and fully immersive systems (e.g., HMD). Additionally, there were no limitations regarding the assessment or intervention administration, frequency, duration, or intensity or sessions.

Articles were excluded if they did not provide outcome data from objective clinical measures (such as cognitive and motor assessment instruments, questionnaires, interviews), were not peer-reviewed or were systematic reviews or meta-analyses. In addition, articles known by the authors from fulfilling the search criteria but not accessible through the search above were also added to the review. These articles were all published in the International Conference on Disability, Virtual Reality & Associated Technologies, which does not have its proceedings indexed.

Data from the included articles were extracted by two of the authors (ALF and JL). Inclusion of the articles was discussed and reviewed in two meetings (one in the screening and one in the eligibility phase) with the remaining senior authors. The general characteristics and results of the studies were extracted, namely the author’s name and year of publication, study design, type of participants, targeted domains, type of interaction and display, type of assessment/rehabilitation VR task, outcome measures and main conclusions. These general characteristics are displayed in Tables 1–6.

**Table 1:** Virtual reality-based technologies in a kitchen environment for assessment and rehabilitation of acquired brain injury.

	**Study Design**	**Participants**	**Purpose and targeted domains**	**Interaction, display and level of immersion**	**Assessment/Rehabilitation**	**Outcome Measures**	**Conclusions**
Zhang et al. (2001)	Experimental	30 TBI and 30 volunteers without brain injury	Assessment and Rehabilitation of Executive functions (information processing, problem solving, logical sequencing, and speed of responding)	HMD and touchscreen or computer cursor - Fully Immersive	Daily living tasks were tested and scored in participants using a computer-simulated virtual kitchen. Each subject was evaluated twice within 7 to 10 days.	Instruments: WAIS-Revised; WRAT; WCST; CVLT and FIM. Performance: Score for each subtasks.	A computer-generated VE represents a reproducible tool to assess selected cognitive functions and can be used as a supplement to traditional rehabilitation assessment in ABI.
Hilton et al. (2002)	Non-experimental	7 stroke	Assessment of Executive functions and motor	Screen and a TUI - Semi-Immersive	Coffee preparation: the activity was simulated by toy equivalents, and reproduced in the VE (sequential task).	Performance: Appropriate responses; errors made and nature of errors.	The location of objects, the instructions given, the physical constraints, the ineffective user response, and the visual and auditory feedback were pointed as important aspects when recreating kitchens in VR.
Zhang et al. (2003)	Non-experimental	54 TBI	Assessment of Attention	Screen and mouse - Non-Immersive	Meal preparation both in a VR kitchen and an actual kitchen twice over a 3-week period.	Instruments: WAIS-R. Performance: Time and errors on task completion using VR; real kitchen performance and OT evaluation.	The VR system showed adequate reliability and validity as a method of assessment in persons with ABI.
Edmans et al. (2006)	Experimental	50 stroke	Rehabilitation of Attention	Touch screen - Non-Immersive	Making a hot drink: the errors observed were compared for standardized task performance in the real world and in a VE.	Instruments: SAST; MMSE; Star Cancellation; RBMT; VOSPB; RPAB; TEA; Kimura Box; 10-Hole Peg Test; MI and BI. Performance: Errors and tasks scores.	The results would indicate that this VE may be a useful rehabilitation tool for patients undergoing stroke rehabilitation in hospital Although, it posed a different rehabilitation challenge from the task it was intended to simulate, and so it might not be as effective as intended as a rehabilitation tool.
O’Brien (2007)	Non-experimental	3 stroke	Assessment and Rehabilitation of Levels of engagement	HMD and the investigator (moves the VE obeying the indications of the participant) - Fully Immersive	The participants were asked to perform simple navigation in the most familiar kitchen and then in most unfamiliar. Then, the process is repeated using familiar and non-familiar objects.	Instruments: MI and ITQ. Performance: GSR and Task-Specific Feedback Questionnaire.	Participants' home environments can be simulated for the purposes of post-stroke rehabilitation, but that participants’ personal proclivities might affect successful use of the system.
Edmans et al. (2009)	Experimental	13 stroke	Assessment and Rehabilitation of Attention	Touch screen - Non-Immersive	Intervention phase: prepare a hot drink in a virtual environment. Control phase: attention control training.	Intruments: Barthel ADL score. Performance: Real world hot drink score; Real world hot drink-making errors; Virtual hot drink score.	Trend towards improvement over time in both real and virtual hot drink making ability in both control and intervention phases. No significant differences in the improvements in real and virtual hot drink making ability during all control and intervention phases in the 13 cases.
Cao et al. (2010)	Experimental	7 (4 TBI 2 stroke and 1 meningo-encephalitis) and 13 HC	Assessment of Executive functions	Desktop computer (screen + keyboard and mouse) - Non-Immersive	Using the TVK and after the familiarization session and recall, the assessment session consists in the preparation of a black coffee during 20 minutes.	Instruments: BDVO. Performance: Time; numbers of errors; number of executed steps; omissions.	It is possible to computerize a daily life task (coffee preparation) in the TVK. ABI or HC were able to complete the virtual complex tasks.
Adams et al. (2015)	Non-experimental	14 stroke	Assessment and Rehabilitation of Motor (upper-limb)	Computer desktop and a Kinect TM. - Non-Immersive/Semi-Immersive	In four visits, participants were asked to use their stroke-affected arm to practice a *Meal Preparation* activity that included 17 motorscore sub-tasks.	Instruments: WMFT. Performance: Motor score; shoulder flexion; shoulder abduction; shoulder rotation; forearm rotation.	The results support the criterion validity of VOTA measures as a means of tracking patient progress during an UE rehabilitation program that includes practice of virtual ADLs.
Besnard et al. (2016)	Experimental	19 TBI and 24 HC	Assessment of Executive functions	Desktop computer (screen, keyboard and a mouse) - Non-Immersive	Participants were asked to prepare a cup of coffee in a real context and in the virtual context (NI-VCT).	Instruments: TMT A and B; WCST; TOL; Stroop Test; BADS; Key Search Task; Zoo Map and MSET. Performance: Time to completion in seconds; accomplishment score; total errors; omission errors; commission errors.	TBI participants performed worse than HC on both real and virtual tasks and on all tests of executive functions. Correlation analyses revealed that NI-VCT scores were related to real task scores. The virtual kitchen is a valid tool for IADL assessment in TBI.
Huang et al. (2017)	Non-experimental	8 stroke	Rehabilitation of Motor (upper-limb)	Amadeo (hand rehabilitation robotic device) and Oculus Rift DK2 - Fully Immersive	18 training sessions along six weeks training using the Amadeo (training protocol include 4 different intervention) A: Passive Mode Training B: Assist-as-needed C: 2D VR task-oriented [(1) Flying bird VR game; (2) Spaceship VR game; (3) Transferring VE-simulated supermarket; (4) Transferring VE-kitchen and cooking scenario.] D: 3D VR task-oriented using Oculus Rift DK2 (first-person view SpaceWar RGS)	Instruments: FMA, MAS Performance: active Range of Motion (ROM) and force intensity of fingers	All subacute stroke subjects undergone the proposed rehabilitation approach showed improvement in their motor skills as indicated by clinical evaluation methods using FMA Hand Sub-section and MAS Hand Movement Score, as well as kinematic characteristics suggested by active ROM and output force intensity.
Triandafilou et al. (2018)	Non-experimental	15 stroke	Rehabilitation of Arm movement	Laptop, Xbox Kinect Sensor (Microsoft Corp, Redmond, WA), pen mouse. - Non-Immersive/Semi-Immersive	Virtual Environment for Rehabilitative Gaming Exercises (VERGE): 1) Ball Bump: players move their hands to contact a virtual ball and send it across the table. 2) Trajectory Trace: a user creates a 3D trajectory (black line) and then passes it to another user to erase by retracing. 3) Food Fight: players move their hands to grab food items that they throw at the other avatar. Control: Alice in Wonderland VR (AWVR) and a home exercise program (HEP).	Instruments: VERGE Survey; Hand displacement was measured using a commercial IMU system (Xsens 3D motion tracker system).	85% of the subjects found VERGE an effective means of promoting arm movement. Arm displacement averaged 350m for each VERGE training session. Arm displacement was not significantly less when using VERGE. Participants were split on preference for VERGE, AWVR or HEP. Almost all subjects indicated willingness to perform the training for at least 2–3 days per week at home.
Thielbar et al. (2020)	Non-experimental	20 stroke	Assessment and Rehabilitation of Arm movement	Laptop, Xbox Kinect Sensor (Microsoft Corp, Redmond, WA), pen mouse. - Non-Immersive/Semi-Immersive	VERGE: 1) Ball Bump: players move their hands to contact a virtual ball and send it across the table. 2) Trajectory Trace: a user creates a 3D trajectory (black line) and then passes it to another user to erase by retracing. 3) Food Fight: players move their hands to grab food items that they throw at the other avatar. Control: AWVR and HEP.	Instruments: Hand displacement was measured using a commercial IMU system (Xsens 3D motion tracker system). Performance: exercise time, exercise scores, total hand and shoulder movement.	Mean voluntary hand displacement, after accounting for trunk displacement, was greater than 350 m per therapy session for the VERGE system. Compliance for home-based therapy was 94% of scheduled sessions completed. Multiple players led to longer sessions and more arm movement.

BADS, Behavioral Assessment of the Dysexecutive Syndrome; BDVO, Battery of Visual Decision of Objects; BI, Barthel ADL Index; CVLT, California Verbal Learning Test; FIM, Functional Independence Measure; FMA, Fugl–Meyer Assessment; ITQ, Immersive Tendencies Questionnaire; MAS, Motor Assessment Scale; MI, Motricity Index; MMSE, Mini Mental State Examination; MSET, Modified Six Elements Test; RBMT, Story Recall from Rivermead Behavioral Memory Test; RPAB, Rivermead Perceptual Assessment Battery; SAST, Sheffield Aphasia Screening Test; TEA, Test of Everyday Attention; TMT A and B, Trail-Making Test; TOL, Tower of London; TUI, Tangible User Interface; VOSPB, Visual Object and Space Perception Battery; WAIS-R, Wechsler Adult Intelligence Scale–Revised; WCST, Wisconsin Card Sorting Test; WMFT, Wolf Motor Function Test; WRAT, Wide Range Achievement Test-3rd Edition.

**Table 2:** Virtual reality-based technologies in a supermarket environment for assessment and rehabilitation of acquired brain injury.

	**Study Design**	**Participants**	**Purpose and targeted domains**	**Interaction, display and level of immersion**	**Assessment/Rehabilitation**	**Outcome Measures**	**Conclusions**
Josman et al. (2006)	Non-experimental	26 stroke	Assessment and Rehabilitation of Executive Functions	Desktop computer (screen + keyboard + mouse) - Non-Immersive	VAPS: purchase seven items from a clearly defined list of products then proceed to the cashier's desk, and pay for them.	Instruments: MMSE and BADS. Performance: Total trajectory, trajectory duration, number of items purchased, number of correct actions, number of incorrect actions, number of pauses, total duration of pauses, and time to pay.	Moderate relationships were found between performance within the VAPS and EF. The results support the use of the virtual supermarket as an EF assessment and training tool after stroke.
Kang et al. (2008)	Experimental	20 stroke	Assessment of Executive Functions, Memory and Attention	HMD and Joystick - Fully Immersive	VR shopping simulation: select and place an item in the cart according to auditory and visual stimulated stimuli, respond to unexpected events such as dropping an item, and select all items in a specified category.	Instruments: K MMSE, K-WAIS; R-KMT, K-FENT and MVPT. Performance: 1. Computer navigation interaction: total time, distance and collision, number of selected items, interaction error and performance index. 2. Memory and attention: immediate and delayed recognition, visual and auditory memory score, attention reaction time, responsiveness, selected items, and attention index. 3. Executive Functions: total time and distance, judgment and calculation score and executive index.	Significant program-performance differences between the stroke and control groups. In the stroke group, decreased perceptive and visuospatial functions might have seriously affected participants’ performance in the VR program.
Raspelli et al. (2012)	Experimental	9 stroke, 10 young and 10 older HC	Assessment of Executive Functions	Screen and joypad - Non-Immersive	VR version of the Multiple Errands Test based on NeuroVR software as an assessment tool for executive functions.	Instruments: MMSE, BIT, Token Test, SCT, Stroop Test, IGT, DEX, TEA, STAI, BDI, ADL and, IADL. Performance: errors, inefficiencies, rule breaks, strategies, interpretation failures, partial task failures.	The construct validity of a virtual version of the Multiple Errands Test has been demonstrated.
Yip and Man (2013)	Experimental	37 ABI	Assessment and Rehabilitation of Prospective Memory	Screen and keyboard or joystick (participants choose the input device they preferred). - Non-Immersive	VRPM: PM training program on everyday PM. Event-based, time-based PM training tasks and ongoing tasks were arranged to occur in a virtual convenience store.	Instruments: MMSE–CV, TONI-3, SADI-CV, CAMPROMPT–CV, HKLLT, FAB, WFT–CV, CTT, CIQ–CV. Performance: VR-based test on everyday PM tasks scores and real life behavioral PM test score.	Significantly better changes were seen in both VR-based and real-life PM outcome measures, related to frontal lobe functions and semantic fluency.
Sorita et al. (2014)	Non-experimental	33 TBI and 17 stroke	Assessment of Executive Functions (planning)	Wall screen, keyboard and mouse - Semi-Immersive	VAPS: participants have to purchase 7 items according to a shopping list.	Instruments: CIQ, CAMPROMPT, POI, WAIS-III, GB and TEA. Performance: Total duration of pauses, total duration of move, total number of errors of selection of items.	Functional performance in VAPS offers promising information on the impact of neuropsychological diseases in daily life.
Josman et al. (2014)	Experimental	24 stroke and 24 HC	Rehabilitation of Action planning, executive functions and IADLs	Desktop computer (screen + keyboard + mouse) - Non-Immersive	VAPS: purchase 7 items from a clearly marked list of products, to then proceed to the cashier’s desk, and to pay for them.	Instruments: BADS and OTDL-R. Performance: Trajectory, trajectory duration, items purchased, correct actions, incorrect actions, number of pauses, duration of pauses and time to pay.	The VAPS showed adequate validity and an ability to predict IADL performance, providing support for its use in cognitive stroke rehabilitation.
Yin et al. (2014)	Experimental	23 stroke	Rehabilitation of Motor: upper-limbs	Sixense and laptop - Semi-Immersive	Virtual local Supermarket: Participants were instructed to pick a virtual fruit from a shelf and release it into a virtual basket as many times as possible within a two minute trial.	Instruments: FMA, ARAT, MAL and FIM.	Although additional VR training was not superior to conventional therapy alone, this study demonstrates the feasibility of VR training in early stroke.
Ogourtsova et al. (2018)	Experimental	27 stroke and 9 HC	Assessment of Unilateral Spatial Neglect	Joystick - Non-Immersive	EVENS consists of two (simple and complex) immersive, 3-D scenes, depicting grocery shopping shelves, with object-detection and navigation tasks.	Instruments: Apples Test, Line Bisection Test, and Star Cancellation Test. Performance: detection time and maximal mediolateral deviation from an ideal navigation trajectory represented by the most direct route possible from the start position to the respective target.	Navigation and detection abilities are affected by environmental complexity of the VR scene in individuals with post-stroke USN and can be employed for USN assessment.
Demers and Levin (2020)	Experimental	22 stroke 15 HC	Assessment of Upper Limbs	Large screen and Kinect II camera - Semi-Immersive	Grocery shopping task with two scenes representing aisles filled with typical supermarket items, to determine whether reach-to-grasp movements made in a low-cost 2D VE were kinematically similar to those made in a physical environment (PE) in healthy subjects and subjects with stroke.	Instruments: CSI; FMA; MoCA; NSA. Performance: Arm and trunk kinematics were recorded with an optoelectronic measurement system (23 markers; 120 Hz). Temporal and spatial characteristics of the endpoint trajectory, arm and trunk movement patterns were compared.	Hand positioning at object contact time and trunk displacement were unaffected by the environment. Compared to PE, in VE, unilateral movements were less smooth and time to peak velocity was prolonged. In HC, bilateral movements were simultaneous and symmetrical in both environments. In subjects with stroke, movements were less symmetrical in VE.
Cogné et al. (2018)	Experimental	40 stroke	Assessment of Executive Functions	Keyboard, mouse and headphones - Non-Immersive	VAPS: purchase 7 items from a list of products, go to the supermarket checkout, pay for the collected items and leave the store in < 30 minutes. There was 5 conditions: 1) without additional auditory stimuli; 2) beeping sounds occurring every 25 sec; 3) sounds from living beings 4) sounds from supermarket objects and; 5) names of other products not in the supermarket.	Instruments: MMSE; GREFEX battery; and Catherine Bergego scale. Performance: Number of products purchased; total time in seconds; and time of navigation.	The 40 stroke patients navigational performance decreased under the 4 conditions with non-contextual auditory stimuli, especially for those with dysexecutive disorders.
Spreij et al. (2020b)	Experimental	88 stroke and 66 HC	Assessment of Executive Functions and Memory	HMD (Oculus Rift or HTC Vive), Computer Monitor and Xbox 360 Controller - Fully-Immersive	The VR task consisted in passing through the entry gates, finding three products from a shopping list, and passing through the cash registers to finish. A grocery list was randomly presented over three trials, and participants were asked to recall the products. There were two different shopping lists: (1) salt, matches, sprinkles; (2) hair wax, cookies, socks.	Instruments: 1 questionnaire regarding user-experience and 1 questionnaire regarding preference. Performance: completion rate, total time needed to complete the VR-task, and total number of products found that were presented on the shopping list.	Both stroke patients and HC reported an enhanced feeling of engagement, transportation, flow, and presence when tested with a HMD but more negative side effects were experienced. The majority of stroke patients had no preference for one interface over the other, yet younger patients tended to prefer the HMD.

ADL, Activities of Daily Living Test; ARAT, Action Research Arm Test; BADS, Behavioral Assessment of Dysexecutive Syndrome; BDI, Beck Depression Inventory; BIT, Behavioral Inattention Test; CAMPROMPT, Cambridge Prospective Memory Test; CAMPROMPT–CV, The Cambridge Prospective Memory Test – Chinese Version; CIQ, Community Integration Questionnaire; CIQ–CV, Chinese Version of the Community Integration Questionnaire; CSI, Composite Spasticity Index; CTT, Color Trails Test; DEX, Dysexecutive Questionnaire; EVENS, Ecological VR-based Evaluation of Neglect Symptoms; FAB, Frontal Assessment Battery; FIM, Functional Independence Measure; FMA, Fugl–Meyer Assessment for the upper extremity; GB, Grober Buschke; HKLLT, Hong Kong List Learning Test; GREFEX, Groupe de réflexion pour l’évaluation des fonctions exécutives; IADL, Instrumental Activities of Daily Living Test; IGT, Iowa Gambling Task; K-FENT, Kim’s Frontal Executive Function Neuropsychological Test; KMMSE, Korean Mini-Intelligence Scale; MAL, Motor Activity Log; MCST, Modified Card Sorting Test; MMSE, Mini Mental State Examination; MMSE–CV, Chinese Version Mini Mental State Examination; MoCA, Montreal Cognitive Assessment; MVPT, Motor-Free Visual Perception Test; NSA, Nottingham Sensory Assessment; OTDL-R, Observed Tasks of Daily Living-Revised; POI, Perceptual Organization Index; R-KMT, Rey-Kim Memory Test; SADI-CV, Chinese Version of the Self-Awareness of Deficit Interview; SCT, Street’s Completion Test; STAI, State and Trait Anxiety Index; Stroop Test, Stroop Colour-Word Test; TEA, Test of Attentional Performance; TMT, Trail Making Test; TONI-3, Test of Nonverbal Intelligence – 3rd Edition; WFT–CV, Word Fluency Test – Chinese Version; WAIS-III, Working Memory Index.

**Table 3:** Virtual reality-based technologies in a shopping mall environment for assessment and rehabilitation.

	**Study Design**	**Participants**	**Purpose and targeted domains**	**Interaction, display and level of immersion**	**Assessment/Rehabilitation**	**Outcome Measures**	**Conclusions**
Rand et al. (2007)	Experimental	14 stroke and 93 HC from three age groups	Assessment and Rehabilitation of Upper Limbs and Executive Functions	GestureTek (IREX system) – Semi-Immersive	VMAll four-item shopping task: the products are located in two different aisles on both the top and middle shelves. The list is written in large letters on a board.	Instruments: MMSE; FMA; SFQ; Borg’s scale of perceived exertion; SFQ- CHILD. Performance: time it took to shop for the four items; the order of products bought while shopping; and the number and type of products bought by mistake.	VMall significantly differentiates between HC and stroke participants. The shopping task was challenging for the stroke participants, which has positive implications for treatment.
Rand et al. (2009a)	Non-experimental	6 stroke	Rehabilitation of Upper Limbs and Executive Functions	GestureTek (IREX system) – Semi-Immersive	Large supermarket in which the users can navigate and select aisles and grocery products by waving the affected hand. Complex, everyday task of shopping in which their weak upper extremity and executive functions deficits can be trained.	Instruments: MMSE; Star Cancellation and GDS. Performance: shopping time and number of mistakes.	VMall has great potential as an intervention tool for treating the weak upper extremity of individuals with stroke while providing opportunities for practicing functional tasks.
Rand et al. (2009b)	Non-experimental	4 stroke	Rehabilitation of Executive Functions	GestureTek (IREX system) – Semi-Immersive	VMall: Virtual supermarket that encourages planning, multitasking, and problem solving while practicing an everyday shopping task. Products are virtually selected and placed in a shopping cart using upper-extremity movements.	Instruments: MET–HV and IADL questionnaire. *Performance:* total number of mistakes; rule break mistakes; non-efficiency mistakes and; use of strategies mistakes.	VMall is a motivating and effective intervention tool for post-stroke rehabilitation of multitasking deficits during the performance of daily tasks.
Rand et al. (2009c)	Experimental	9 stroke, 20 young and 20 older HC	Assessment of Executive Functions	GestureTek (IREX system) – Semi-Immersive	VMall: virtual supermarket that encourages planning, multitasking, and problem solving while practicing an everyday shopping task. Products are virtually selected and placed in a shopping cart using upper-extremity movements.	Instruments: MET–HV; BADS (Zoo map) and IADL questionnaire. Performance: total number of mistakes; rule break mistakes; non-efficiency mistakes and; use of strategies mistakes	VMET differentiates between two age groups of HC and between HC and post-stroke thus demonstrating that it is sensitive to brain injury and ageing. Significant correlations were found between MET and VMET for both post-stroke and older HC.
Hadad et al. (2012)	Experimental	5 stroke and 6 HC	Assessment and Rehabilitation of Executive Functions	Kinect (SeeMe system) – Semi-Immersive	VIS: a VE of a 3-store mall. A store mock-up. A Cafeteria that served as location of shopping in a real setting.	Instruments: MMSE; FIM; SFQ. Performance: time to complete the test and the number of errors (buying the wrong item or not buying an item on the list). Modified version: budget handling.	Post-stroke group performed more slowly than control group. Both groups took longer to complete the test in the VIS than in the store mock- up and the cafeteria. Performance in the VIS, the store mock-up and the cafeteria were correlated in the post-stroke group. The VIS may be used to assess and train shopping cognitive abilities.
Jacoby et al. (2013)	Experimental	12 TBI	Assessment of Executive Functions	GestureTek (IREX system) – Semi-Immersive	VMall: a supermarket that enables training of motor and cognitive abilities during IADL practice. It enables the performance of a variety of tasks.	Instruments: MET; EFPT. Performance: Total score.	Results show a trend towards an advantage of VR therapy compared to cognitive retraining OT without VR, as it leads to greater improvement in IADL’s.
Erez et al. (2013)	Experimental	20 TBI children and 20 typically developing children	Assessment and Rehabilitation of Executive Functions	GestureTek (IREX system) – Semi-Immersive	VMall: a supermarket that enables training of motor, cognitive and EF abilities during IADL practice. It enables the performance of a variety of tasks.	Instruments: SFQ-CHILD; Borg’s scale of perceived exertion; BADS (Zoo Map). Performance: Time to complete; number of mistakes (items bought by mistake, additional items bought, or required items not bought); and sequence of bought products.	Good usability. Significant difference in performance between the two groups: mean shopping time and number of mistakes higher for TBI children. Poorer performance of TBI children may be due to EF deficits.
Okahashi et al. (2013)	Experimental	5 Stroke, 5 TBI, 10 HC, 10 old and 10 young HC.	Assessment of Executive Functions, Attention and Memory	Touch panel display: by touching the bottom of the screen, users could move in the virtual shopping mall, entering a shop and buying an item. – Semi-Immersive	We set a shopping task, which asks users to buy four specific items. The user must search the shops that sell specific items and select the target item out of six items inside a shop.	Instruments: MMSE; RBMT; EMC; DEX; SDMT; SRT; Star and Letter Cancellation Task. Performance: Bag use; list use; cue use; forward movement; reverse movement; correct purchases; total time; time in shops; time on road and mean time per shop.	VST is able to evaluate the ability of attention and everyday memory in participants with brain damage. The time of VST is increased by age.
Canty et al. (2014)	Experimental	30 TBI and 24 HC	Assessment of Prospective Memory	Keyboard, computer. – Non-Immersive	VRST: simulates the graphical perspective of the participant’s avatar and requires the participant to purchase 12 items in a pre-specified order from a selection of 20 shops. Participants were instructed to complete 3 time- and 3 event-based PM tasks.	Instruments: LDPMT; TMT A and B; HVLT-R; COWAT; LNS; HSCT; SPRS; IR; IL. Performance: Performance in cue detection.	The performance of the TBI group was significantly poorer than that of controls on event-based PM, as measured by the LDPMT, and on time- and event-based PM as measured by the VRST.
Nir-Hadad et al. (2015)	Experimental	19 stroke and 20 HC	Assessment of Executive Functions	Kinect (SeeMe system) – Semi-Immersive	Adapted Four-Item Shopping Task in the SeeMe VIS environment with a supermarket, a toy store and a hardware store.	Instruments: SFQ; BADS (Zoo Map and the Rule Shift Cards); EFPT (Telephone Use and Bill Payment); FIM; FMA; CDT. Performance: selected products; selection time; mistakes; total cost of purchased items; and distance traversed while shopping.	Good initial support for the validity of the Adapted Four-Item Shopping Task as an IADL assessment that requires the use of EF.

BADS, Behavioral Assessment of the Disexecutive Syndrome; Borg’s scale of perceived exertion; CDT, Clock Drawing Test; COWAT, Controlled Oral Word Association Task; DEX, Dysexecutive Questionnaire; EFPT, Executive Function Performance Test; EFPT, Executive Function Performance Test; EMC, Everyday Memory Checklist; FIM, Functional Independence Measure; FMA, Fugl–Meyer Motor Assessment; GDS, Geriatric Depression Scale; HSCT, Hayling Sentence Completion Test; HVLT-R-TR, Total Recall on the Hopkins Verbal Learning Test-Revise; IADL Performance, Instrumental Activities of Daily Living Performance; IADL questionnaire, Instrumental Activities of Daily Living questionnaire; IL, independent living; IR, interpersonal relationships; LDPMT, Lexical decision prospective memory task; LNS, Letter Number Sequencing; MET, Multiple Errands Test; MET–HV, Multiple Errands Test–Hospital Version; MET-SV, Multiple Errands Test—Simplified Version; MMSE, Mini-Mental State Examination; RBMT, Rivermead Behavioral Memory Test; SDMT, Symbol Digit Modalities Test; SFQ-CHILD, Short Feedback Questionnaire for children; SFQ, Short Feedback Questionnaire; SPRS, Sydney Psychosocial Reintegration Scale; SRT, Simple Reaction Time Task; TMT, Trail Making Test: Parts A and B.

**Table 4:** Virtual reality-based technologies in a street environment for assessment and rehabilitation.

	**Study Design**	**Participants**	**Purpose and targeted domains**	**Interaction, display and level of immersion**	**Assessment/Rehabilitation**	**Outcome Measures**	**Conclusions**
Naveh et al. (2000)	Experimental	6 HC and 6 stroke with USN	Assessment and Rehabilitation of Attention, Street crossing consciousness.	15” CRT monitor or projected on a screen via video projector, keyboard and mouse. - Semi-Immersive	The participants’ task was to commence crossing the street when, in his or her opinion, it was safe to do so. The number of training sessions varied from 1 to 4, and the duration of each session varied from 30 to 60 minutes.	Instruments: BIT; MSC and ADL’s questionnaire. Performance: Frequency, order and direction that participants searched for oncoming vehicles; number of trials and total time to successfully complete each level; highest level successfully completed; time taken at each level of difficulty to cross the virtual street safely; number of accidents at each level; number of times looking to the left and to the right before crossing the street.	The performance of the patients was considerably more variable; they were able to complete fewer levels and usually took more time to do so. The results indicate that the VR training could be beneficial to people who have difficulty with street crossing.
Wald et al. (2000)	Non-experimental	28 TBI	Assessment of Perception (drive performance)	DriVR and HMD - Fully Immersive	Concurrent validity of the DriVR was examined by comparing DriVR measures to other indicators of driving ability, which consisted of on-road, cognitive and visual-perceptual, and driving video tests. The entire driving assessment occurred over a 2-day period within the same week.	Instruments: DPT II; DRI II and On-road Driving test. Performance: Follow traffic event; shop road and opposite road; stop signs missed; driveway choice event and avoid traffic event.	The DriVR appeared to be a useful adjunctive screening tool for assessing driving performance in persons with ABI.
Weiss et al. (2003)	Experimental	6 stroke with USN and 6 HC	Assessment and Rehabilitation of Attention, Street crossing consciousness.	Screen, Keyboard and mouse - Non-Immersive	The subject’s task was to commence crossing the street when, in his or her opinion, it was safe to do so. In the initial study, the number of training sessions varied from 1 to 4. During the intervention study, there were 12 training sessions given over a period of four weeks. In each configuration, the levels of difficulty were graded from 1 to 7.	Performance: Frequency, order and direction searching for oncoming vehicles; number of trials as well as the total time it took to successfully complete each level; highest level successfully completed; time taken at each level to cross the virtual street safely; number of accidents at each level; number of times looking to the left and to the right before crossing the street.	This VE was suitable in both its cognitive and motor demands for the targeted population. The VR training is likely to prove beneficial to people who have difficulty with crossing streets.
Katz et al. (2005)	Experimental	19 stroke participants with USN	Assessment of Attention, street crossing consciousness.	Computer desktop, keyboard and mouse - Non-Immersive	Experimental group was given computer based VR street crossing training and control group was given computer based visual scanning tasks, both for 9 hours distributed in twelve sessions for four weeks.	Instruments: BIT; MSC and ADL checklist. Performance: Number of looks to the left; number of looks to the right and number of accidents.	Both groups improved similarly. The VR group achieved on the USN measures results that equaled those achieved by the control group treated with conventional visual scanning tasks.
Titov and Knight (2005)	Non-Experimental	3 participants with neurological injuries	Assessment of Prospective memory	Touchscreen connected to a laptop - Non-Immersive	Virtual Street: a street that participants can ‘walk’ along. It has distracting sounds (car horns, people talking, music, news reports). Animations, such as people walking on the scene have been added. Prospective memory can be tested using the Virtual Street environment by instructing participants to complete errands as they walk along.	Instruments: WMS-III Digit Span; NART Word Lists; WCST; F-A-S Verbal Fluency and Stroop Test. Performance: SMT and CMT.	The patient performed more poorly on the multi-tasking test than the matched control. Patients performance in the computer-based tests was consistent with their memory deficits.
Lloyd et al. (2006)	Non-Experimental	20 ABI and 14 HC	Assessment and Rehabilitation of Route Memory	Playstation 2 games console and TV - Non-Immersive	Driv3r is based upon real-world town of Nice, and contains a large network of streets and buildings. It also features drivers and pedestrians moving around. Study 1: HC were driven around the real street and then had to give directions to the driver in the virtual street and contrariwise in a counterbalanced order. Study 2: Participants learned one route in an errorless way, watching the entire route correctly through completion three times before attempting to call out directions themselves. Another route, they learned in an errorful way, being shown the correct route only once before being asked to take two practice attempts (during which errors typically occurred) at calling out the directions before the final, ‘test’ trial.	Performance: number of wrong turns, number of errors, used strategies.	The first study demonstrated the ecological validity of a non-immersive virtual town, showing performance therein to correlate well with real-world route learning performance. The second study found that a rehabilitation strategy known as ‘errorless learning’ is more effective than traditional ‘trial- and-error’ methods for route learning tasks.
Kim et al. (2007)	Experimental	10 stroke and 40 HC	Rehabilitation of Unilateral neglect	HMD and Mouse - Fully Immersive	The training procedure consisted on completing missions while keeping the virtual avatar safe during crossing the street in a VE.	Instruments: MMSE; line bisection and cancellation test. Performance: deviation angle; reaction time; right and left reaction time; visual cue; auditory cue; failure rate of mission.	The system was proper to the training of unilateral neglect patients.
Akinwuntan et al. (2009)	Experimental	73 stroke	Rehabilitation of Cognitive training	Driving simulator - Semi-Immersive	Forty-two participants received simulator-based driving training, whereas 41 participants received cognitive training for 15 hours.	Instruments: CARA.	Simulator-based driving training improved driving ability, especially for well educated and less disabled stroke patients.
Devos et al. (2009)	Experimental	73 stroke	Rehabilitation of Cognitive training (driving retraining)	Driving simulator - Semi-Immersive	Forty-two participants received simulator-based driving training, whereas 41 participants received cognitive training for 15 hours.	Instruments: TRIP; CARA.	Contextual training in a driving simulator appeared to be superior to cognitive training to treat impaired on-road driving skills after stroke.
Lloyd et al. (2009)	Experimental	20 ABI (8 TBI, 6 stroke, 6 others (brain tumor and cortical cysts)	Assessment and Rehabilitation of Learning (Route learning)	Screen and Ps2 - Non-Immersive	For the demonstration trial and two learning trials, participants were shown around a route. As in the errorless condition, participants were asked to watch as the experimenter moved along the route and called out directions in the demonstration trial. For the test trial participants were asked to call out the directions at each junction.	Instruments: ROCF; AMIPB (List Learning). Performance: Wrong turns and errors made.	Route recall following the errorless learning was significantly more accurate than recall after errorfull learning. This suggests that the benefits of errorless over errorfull learning in ABI rehabilitation extend beyond verbal learning tasks to the route memorization task.
Sorita et al. (2013)	Non-experimental	27 TBI	Assessment of Learning (route learning)	Screen and joystick - Non-Immersive	Two immediate and one delayed route recall assessing procedural learning in spatial memory. The two tasks were performed within two modalities: route learning within a RE district and route learning within a virtual reproduction of the urban district (VE).	Instruments: GCS; TEA and GBT. Performance: Sketch map test; Map recognition test; Scene arrangement test; VE design.	The routes learned within the VE transferred better to the real setting than the routes directly learned in the RE. Therefore VR might provide ecological rehabilitation scenarios to assess daily functioning.
Navarro et al. (2013)	Experimental	32 stroke (15 non-USN and 17 USN) and 15 HC	Assessment of Arousal, Attention, Consciousness. (Street crossing).	Screen, infrared tracking system and joystick - Non-Immersive	The assessment session consisted of two consecutive repetitions of virtual street crossing. In each session, the participants were asked to move from the starting point to a large department store and then to come back as quickly and safely as possible.	Instruments: BIT; CPT-II; Stroop Test; CTT; BADS (Zoo Map and Key Search Test). Performance: Time to complete the task; number of head turns, accidents and warning signs.	The performance of neglect participants was significantly worse than the performance of non-neglect and HC.
Park (2015)	Experimental	30 stroke	Assessment of Incidence of driving errors	Driving simulator (GDS-300, Gridspace) - Semi-Immersive	Virtual on-road course with road traffic rules. The test course simulated driving in downtown Seoul and on the highway and was designed to resemble actual driving, incorporating various buildings, moving cars, traffic signals, and road signs.	Performance: Failure to use seat belt; exceed speed limit; turn signal errors; drop out the course; cross center line; accidents; brake reaction time and total error score.	Patients with lesions in the left or right hemispheres showed differences in driving skills, such as: frequencies of center line crossing, turn signal errors, accidents, brake reaction time, total driving errors and test failure rate.
Ettenhofer et al. (2019)	Experimental	17 TBI	Assessment and Rehabilitation of Dual processing, attention, working memory, and response inhibition	General Simulation Driver Guidance System: 8-foot circular frame supporting a curved screen and a driving console analogous to that found in a typical automobile - Semi-Immersive	1) Brief review of training and progress; 2) practice of cognitive skills through cognitive driving scenarios; 3) practice of composite driving while performing working memory or visual attention tasks; and 4) open-ended race-track course to promote engagement.	Instruments: WAIS-III (Digit Span, Symbol Search, Coding); TMT A and B; Letter and Animal Fluency; CVLT-II; Grooved Pegboard; NSI; PTSD Checklist-Civilian; BDI-II; SWLS; SF-36 (physical and mental); ESS and FSS. Performance: VR Tactical and Operational Driving Quotient.	NeuroDRIVE intervention enabled significant improvements in working memory and selective attention.
Spreij et al. (2020a)	Experimental	138 stroke and 21 HC	Assessment of Visuo Spatial Neglect	Large Screen + steering wheel - Semi-Immersive	Participants were instructed to use the steering wheel to maintain the starting position at the center of the right lane, which is in line with Dutch road traffic regulations. Participants needed to adjust their position continuously.	Instruments: Shape Cancellation Task; Catherine Bergego Scale. Performance: Average position on the road and the average standard deviation of the position, as an indication of the magnitude of sway.	Patients with left-sided VSN and recovered VSN deviated more regarding position on the road compared to patients without VSN. The deviation was larger in patients with more severe VSN. Regarding diagnostic accuracy, 29% of recovered VSN patients and 6% of patients without VSN showed abnormal performance on the simulated driving task. The sensitivity was 52% for left-sided VSN.
Wagner et al. (2021)	Non-experimental	18 stroke (9 USN and 9 No USN)	Assessment of Unilateral Spatial Neglect	iVRoad HTC Vive Controller - Fully Immersive	The task consists of dropping a letter in a mailbox on the way to work. To do so, the user first has to safely cross two roads and the square in between, and then return to the starting position to continue his/her way to work.	Instruments: IPQ; USEQ; SSQ. Performance: Error rate; decision time (elapsed time between task onset and start of road crossing by button press); Head direction ratio between the time patients looked left and right before making the decision to cross the street.	Parameters and conditions for distinguishing patients with and without USN were identified: Decision time has been identified as a very good measure. Error rate and the Head direction ratio were also reliable measures of separation.

ADL checklist, Activities of daily living checklist; AMIPB, Adult Memory and Information Processing battery; BADS, Behavioral Assessment of the Dysexecutive Syndrome; BDI-II, Beck Depression Inventory; BIT, Behavioral Inattention Test; CARA, Center for Fitness to Drive Evaluation and Car Adaptations; CMT, Complex Multi-tasking Test; COWAT, Controlled Oral Word Association Task; CPT-II, Conner’s Continuous Performance Test-II; CTT, Color Trail Test; CVLT-II, California Verbal Learning Test II; D-KEFS, Delis–Kaplan Executive Function System; DPT-II, Driver Performance Test II; DRI-II, Driver Risk Index II; ESS, Epworth Sleepiness Scale; FAS, FAS Verbal Fluency Test; FSS, Fatigue Severity Scale; GBT, Grober and Buschke’s test; GCS, Glasgow Coma Score; IPQ, Igroup Presence Questionnaire; MRMTDS, Money Road Map Test of Direction Sense; MSC, Mesulam Symbol Cancellation test; NART, National Adult Reading Test; NSI, Neurobehavioral Symptom Inventory; ROCF, Rey Osterrieth Complex Figure Task; SMT, Simple Multi-tasking Test; SSQ, Simulator Sickness Questionnaire; SWLS, Satisfaction with Life Scale; TEA, Test for Assessment of Attention; TMT, Trail Making Test; TRIP, Test Ride for Investigating Practical fitness to drive; USEQ, User Satisfaction Evaluation Questionnaire; VSN, Visuo Spatial Neglet; WAIS, Wechsler Adult Intelligence Scale; WCST, Wisconsin Card Sorting Test; WMS III, Wechsler Memory Scale III.

**Table 5:** Virtual reality-based technologies in a city environment for assessment and rehabilitation of acquired brain injury.

	Methods/Study design	Participants	Domain	Interaction and display/Instrumentation	Assessment/Treatment	Measures	Conclusions
Gamito et al. (2011a,b)	Non-experimental	1 TBI	Working Memory and Attention.	HMD, keyboard and mouse.	Small town populated with digital robots. The town comprised several buildings arranged in eight squared blocks, along with a 2-room apartment and a mini-market, where the participant was able to move freely around and to grab objects.	Instruments: PASAT. Performance: Completion time of each task as an indicator of task performance speed.	Satisfactory level of performance after some practice, with an average time for each task of 5 min. These data revealed a significant increase in working memory and attention levels, suggesting an improvement on patient cognitive function.
Gamito et al. (2011a,b)	Non-experimental	2 stroke	Memory and Attention.	HMD, keyboard and mouse.	Activities of daily living such as: morning hygiene, taking the breakfast, finding the way to the minimarket and buying several items from a shopping list.	Instruments: WMS-III; TP; PQ; Immersion and Cybersickness. Performance: Completion time of each task as an indicator of task performance speed.	Increase in memory and attention/concentration skills, which can suggest a higher level of executive functioning after the VR training.
Jovanovski et al. (2012)	Experimental	11 stroke, 2 TBI and 30 HC	Evaluate the ecological validity of the MCT and other standardized executive function tests.	Joystick and computer screen	The MCT consists of a post office, drug store, stationery store, coffee shop, grocery store, optometrist’s office, doctor’s office, restaurant and pub, bank, dry cleaner, pet store, and the participant’s home.	Instruments: WTAR; TOMM; COWAT; WCST; BADS-MSE; TMT A and B; WAIS-III (Block Design, and Digit Span); JLO; ROCF; CVLT-II; WMS-III (Logical Memory); BDI-II and BAI. Performance: Completion Time; number of Tasks Completed; and Task Repetitions. Qualitative errors: Task Failures, Task Repetitions, or Inefficiencies.	VR technology, designed with ecological validity in mind, are valuable tools for neuropsychologists attempting to predict real-world functioning in participants with ABI.
Gamito et al. (2014)	Non-experimental	17 stroke	Memory and attention	HMD, desktop screenplay, keyboard and mouse.	Small town with a 2-room apartment and a minimarket in the vicinity.	Instruments: WMS-III; ROCF; TP. Performance: a therapist assessed the session outcome.	Increased working memory and sustained attention from initial to final assessment regardless of the VR device used.
Vourvopoulos et al. (2014)	Non-experimental	7 stroke, 2 TBI and 1 MCI	Visuospatial orientation, Attention and Executive functions.	Joystick and computer screen	Simulations of several activities of daily living (supermarket, post-office, pharmacy and bank) within a city.	Instruments: MMSE; SIS and SUS. Performance: Total score and execution time.	Strong correlation between the Reh@City performance and the MMSE score. High usability scores (*M* = 77%).
Gamito et al. (2015)	Experimental	20 stroke	Memory and Attention	HMD, keyboard and mouse.	The VR scenario comprised several daily life activities: buying several items, finding the way to the minimarket, finding a virtual character dressed in yellow, recognition of outdoor advertisements and digit retention.	Instruments: WMS-III; ROCF; TP.	Significant improvements in attention and memory functions in the intervention group, but not in the controls.
Faria et al. (2016)	Experimental	18 stroke	Global cognitive functioning	Joystick and computer screen	Reh@City v1.0: an immersive three-dimensional environment with streets, sidewalks, commercial buildings, parks and moving cars. Participants have to accomplish some common ADL’s in four places: supermarket, post office, bank, and pharmacy.	Instruments: ACE; TMT A and B; WAIS III (Picture Arrangement); SIS 3.0; SUS. Performance: Total score and execution time.	A within groups analysis revealed significant improvements in global cognitive functioning, attention, memory, visuo-spatial abilities, executive functions, emotion and overall recovery in the VR group. The control group only improved in self-reported memory and social participation. Between groups analysis, showed significantly greater improvements in global cognitive functioning, attention and executive functions when comparing VR to conventional therapy.
Claessen et al. (2016)	Experimental	68 stroke and 44 HC	Spatial navigation	Not defined.	Each route contained 11 decision points. Eight subtasks were used to assess the participants’ knowledge of the studied route in the testing phase.	Instruments: NART; CORSI; TMT A and B; WAIS-III (Digit Span). Performance: Scene Recognition.	Moderate overlap of the total scores between the two navigation tests indicates that virtual testing of navigation ability is a valid alternative to navigation tests that rely on real-world route exposure.
Faria et al. (2019)	Experimental	31 stroke	Global cognitive functioning	Customized handle with a tracking pattern.	Reh@City v2.0: an immersive three-dimensional environment with streets, sidewalks, commercial buildings, parks and moving cars. Participants have to accomplish some common ADL’s in eight places: supermarket, post office, bank, pharmacy, fashion store, kiosk, home and park.	Instruments: MoCA. Performance: Total score and execution time.	Both groups performed at the same level and there was not an effect of the training methodology in overall performance. However, Reh@City enabled a more intensive training, which may translate in more cognitive improvements.
Faria et al. (2020)	Experimental	36 stroke	Global cognitive functioning	Customized handle with a tracking pattern.	Reh@City v2.0: an immersive three-dimensional environment with streets, sidewalks, commercial buildings, parks and moving cars. Participants have to accomplish some common ADL’s in eight places: supermarket, post office, bank, pharmacy, fashion store, kiosk, home and park.	Instruments: MoCA; TMT A and B; WMS-III Verbal Paired Associates; WAIS-III Digit Symbol Coding, Symbol Search, Digit Span and Vocabulary subtests, and PRECiS.	Within-groups Reh@City v2.0 improved general cognitive functioning, attention, visuospatial ability and executive functions. These improvements generalized to verbal memory, processing speed and self-perceived cognitive deficits assessments. TG only improved MoCA orientation, and processing speed and verbal memory outcomes. Between-groups Reh@City v2.0 was superior in MoCA general cognitive functioning, visuospatial ability, and executive functions.
Oliveira et al. (2020)	Experimental	30 stroke	Global cognitive functioning	Mouse and computer screen	City with tasks as using a toothbrush, following the steps of a recipe to bake a cake, or by watching news on a television and recollect pieces of this information on latter tasks. Outside the tasks consist of buying products in the grocery store or to perform visual search tasks in a virtual art gallery.	Instruments: MoCA, FAB, WMS-III, and CTT.	Improvements were found in the assessed cognitive domains at 6 to 10 post-treatment sessions. In-depth analysis through reliable change scores has suggested larger treatment effects on global cognition.

ACE, Addenbrooke Cognitive Examination; BADS-MSE, Behavioral Assessment of the Dysexecutive Syndrome–Modified Six Elements Test; BAI, Beck Anxiety Inventory; BDI-II, The Beck Depression Inventory-Second Edition; CORSI, The Corsi Block-Tapping Task; COWAT, Controlled Oral Word Association Test; CTT, Colors Trail Test; CVLT-II, California Verbal Learning Test-Second Edition; Cybersickness, Simulator Sickness Questionnaire; FAB, Frontal Assessment Battery; Immersion, Immersion Tendencies Questionnaire; JLO, Judgment of Line Orientation; MMSE, Mini-Mental State Examination test; MoCA, Montreal Cognitive Assessment; NART, National Adult Reading Test; PASAT, The Paced Auditory Serial Addition Task; PQ, Presence Questionnaire; PRECiS, Patient-Reported Evaluation of Cognitive State; ROCF, Rey-Osterreith Complex Figure Test; SIS, Stroke Impact Scale 3.0; SUS, System Usability Scale; TMT A and B, Trail-Making Test Parts A and B; TOMM, Test of Memory Malingering; TP, Toulouse–Pieron; WAIS-III, Wechsler Adult Intelligence Scale-Third Edition; WCST, Wisconsin Card-Sorting Test; WMS-III, Wechsler Memory Scale – 3rd edition; WTAR, The Wechsler Test of Adult Reading.

**Table 6:** Virtual reality-based technologies in other everyday life environments for assessment and rehabilitation of acquired brain injury.

	Methods/Study design	Participants	Domain	Interaction and display/Instrumentation	Assessment/Treatment	Measures	Conclusions
Matheis et al. (2007)	Experimental	20 TBI and 20 HC	Learning and Memory	Dell laptop +5TH Dimension Technologies (5DT) 800 Series HMD	The participant had to learn 16 target items, depicted among numerous other office distracters (e.g., computer, file cabinet), during a sequence of 12 sequential exposures to the VR Office environment.	Instruments: WASI; WAIS (digit span and digit symbol-coding subtests); TMT A and B; WCST; BNT; HVOT; COWAT; CVLT; BVMT-R; and m-SSQ. Performance: Initial list acquisition, short-term recall (30 min.) and long term recall (24 h).	VR memory testing accurately distinguished the TBI group from controls. Non-memory-impaired TBI acquired targets at the same rate as HC. There was a relationship between the VR Office and a standard measure of memory, suggesting the construct validity of the task.
Fong et al. (2010)	Non-experimental	12 intracerebral hemorrhage, 6 stroke, 5 TBI and 1 brain tumor	Cognition	27″ touch monitor that represents the actual dimensions of a real ATM.	VR-ATM	Instruments: MMSE and COGNISTAT. Performance: Success or failure in using VR-ATM real ATM (cash withdrawals, money transfers, and electronic payments); average reaction time; percentage of incorrect responses; number of cues needed; and time spent.	Sensitivity was 100% for cash withdrawals and 83.3% for money transfers. Specificity was 83 and 75%, respectively. For cash withdrawals, average reaction time of the VR group was shorter than the conventional program group. No differences in average reaction time or accuracy between groups for money transfers, although there was improvement for the VR-ATM group.
Renison et al. (2012)	Experimental	30 TBI and 30 HC	Executive Functions	An X-box and Playstation compatible handset.	VLT models the dimensions and contents of two rooms in the Library at Epworth Hospital. Participants perform several tasks associated with the day to day running of the library.	Instruments: BADS Zoo Map and MSET; WTAR; WMS III; WAIS III (Digit span); WCST; BSAT and DEX. Performance: Task analysis, strategy generation and regulation, prospective working memory, interference and dual task management, response inhibition, time-based prospective memory and event-based prospective memory tasks scores.	Performances on the VLT and the RLT were correlated indicating that VR performance is similar to real world performance. TBI group performed significantly worse than the control group on the VLT and the MSET but the other four measures of EF failed to differentiate the groups. Both MSET and VLT predicted everyday EF suggesting that are both ecologically valid tools for EF assessment. VLT has the advantage of providing objective measurement of EF individual components.
Krch et al. (2013)	Non-experimental	7 TBI, 5 MS and 7 HC	Executive Functions	Computer and mouse	Assessim Office: respond to emails; decide whether to accept or reject real estate offers based on specific criteria; print the real estate offers that met specific criteria; retrieve printed offers from the printer and deliver them to a file box located on participants’ desk; and ensure that the conference room projector light remained on.	Instruments: WAIS III; D-KEFS; WASI Performance: emails correctly replied; correct decision, real estate offers; declined offers incorrectly printed; printed offers delivered to file box; projector light missed; redundant clicks.	AO was well tolerated by TBI and MS samples. Performance by clinical samples on the AO was distinct from HC. Patient performance was poorer than HC across all AO tasks. Evaluation of the relationship between performance on AO tasks and EF tests revealed that there were more significant relationships within TBI group as compared with MS group.
Fluet et al. (2013)	Experimental	30 stroke	Upper-limbs	Computer and CyberGlove: Subject interacted with VE with haptic guidance provided by robot.	VE activities: Reach/Touch; Placing Cups; Hammer Task; Blood Cell; Plasma Pong; Hummingbird Hunt; Piano Trainer and; Space Pong.	Instruments: UEFMA; WMFT; JTHF. Performance: Simulations and real activities performance.	Both groups improved in UEFMA, WMT and JTHF. Gains in UEFMA maintained at follow-up. No differences at any of the three measurement times and no significant group time interactions.
Gerber et al. (2014)	Non-experimental	19 TBI	Engagement in computer-based simulations of functional tasks.	Computer and a haptic device: Phantom® OmniTM.	Interactive virtual scenes in 3D space: remove tools from a workbench, compose 3 letter words, manipulate utensils to prepare a sandwich, and tool use.	Instruments: WMFT; BPS; NSI and PPT. Performance: *Tool use*: grabbing the tool; tool interaction with nail or screw; movement of nail or screw; and success in completing the task. *Making a sandwich*: grabbing an object (piece of bread or knife); moving bread; touching the jar cover; touching peanut or jelly or bread with the knife. *Spelling:* grabbing and releasing a letter; placing letter on the grid; and completing a three-letter word.	Participants reported being engaged in using haptic devices that interact with 3D VEs. Haptic devices are able to capture objective data about motor and cognitive performance.
Gilboa et al. (2017)	Experimental	29 ABI children and adolescents and 30 HC	Executive Functions	Computer and mouse	JEF-C: the participant has to plan, set up and run its own party through the completion of tasks.	Instruments: WASI, BADS-C and the BRIEF questionnaire (parents). Performance in 8 different cognitive constructs: planning; prioritization; selective, adaptive and creative thinking and; action, time and event-based prospective memory.	Patients performed significantly worse than controls on most of the JEF-C subscales and total score, with 41.4% of participants with ABI classified as having severe executive dysfunction.
Cho and Lee (2019)	Experimental	42 stroke	Cognitive functioning	HMD developed by Company S	Fishing: the user catches fish using upper extremities. Picture matching: the user flips cards and finds a match, the initial screen has 8 cards; the user can turn or look back to see all the cards. The user needs to place his or her hand on the card whose picture they want to check as if they reach out and touch it. RehaCom	Instruments: CNT, LOTCA, FIM.	Virtual reality immersive training might be an affordable approach for cognitive function and activity of daily living performance recovery for patients with acute stroke.
Qiu et al. (2020)	Non-Experimental	15 stroke	Upper-extremities	Laptop and Leap Motion Controller (with arm supporters)	HoVRS: 5 games (Maze, Wrist Flying, Finger Flying, Car, Fruit Catch) targeting different movement patterns (Elbow-Shoulder, Wrist, Hand, Whole Arm)	Intruments: UEFMA Performance: six hand and arm kinematic outcomes (HOR, WPR, HRR, HOA, WPa, HRA)	Subjects spent 13.5 h using the system at home and demonstrated an increase of 5.2 on the UEFMA, which exceeds the minimally clinically important difference of 4.25. They also improved in six measurements of hand kinematics.
Lorentz et al. (2021)	Non-experimental	21 stroke, 6 TBI, 6 degenerative disease, 1 encephalitis and 1 brain tumor	Attention and Working Memory	Oculus quest with two OLED displays and two touch-controllers.	VR Traveller: each scenario is set in a different location and challenges alertness, selective attention, visual scanning and working memory. For instance, the New York City module primarily trains tonic alertness: patients are asked to press a response key as soon as a skyscraper lights up. At higher levels, visual distractions (streets, cars, and traffic lights) are added in order to place an additional cognitive load on patients’ selective attention.	Instruments: UEQ, a self-constructed feasibility questionnaire, and the VRSQ.	Patients’ ratings of the VR training in terms of acceptability and feasibility were positive.

BADS, Behavioral Assessment of the Dysexecutive Syndrome; BPS, Boredom Propensity Scale; BSAT, Brixton Spatial Anticipation Test; BNT, Boston Naming Test; BRIEF, Behavior Rating Inventory of Executive Function; BVNTR, Brief Visuospatial Memory Test-Revised; CNT, Computerized Neurocognitive Function Test; COGNISTAT, The Neurobehavioral Cognitive Status Examination; COWAT, Controlled Oral Word Association Test; CVLT, California Verbal Learning Test; DEX, Dysexecutive Questionnaire; D-KEFS, Delis–Kaplan Executive Function System; FIM, Functional Independence Measure; HMD, Head Mount Display; HoVRS, Home based Virtual Rehabilitation System (HoVRS); HVOT, Hooper Visual Organization Test; JEF-C, Jansari assessment of Executive Functions for Children; JTHF, Jebsen Test of Hand Function; LOTCA, Loewenstein Occupational Therapy Cognitive Assessment; MMSE, Mini-Mental State Examination; MSET, Modified Six Elements Test; m-SSQ, modified Simulator Sickness Questionnaire; NSI, Neuropsychological symptom inventory; PPT, Purdue Peg Motor Test; TBI, Traumatic Brain Injury; TMT, Trail Making Test; UEFMA, Upper Extremity Fugl Meyer Assessment; UEQ, User Experience Questionnaire; VRSQ, Virtual Reality Sickness Questionnaire; WAIS-III, Wechsler Adult Intelligence Scale-Third Edition; WASI, Wechsler Abbreviated Scale of Intelligence; WCST, Wisconsin Card Sorting Test; WMFT, Wolf Motor Function Test; WMS-III, Wechsler Memory Scale – Third Edition; WTAR, Wechsler Test of Adult Reading.

## 3. Results

**Figure 1:**
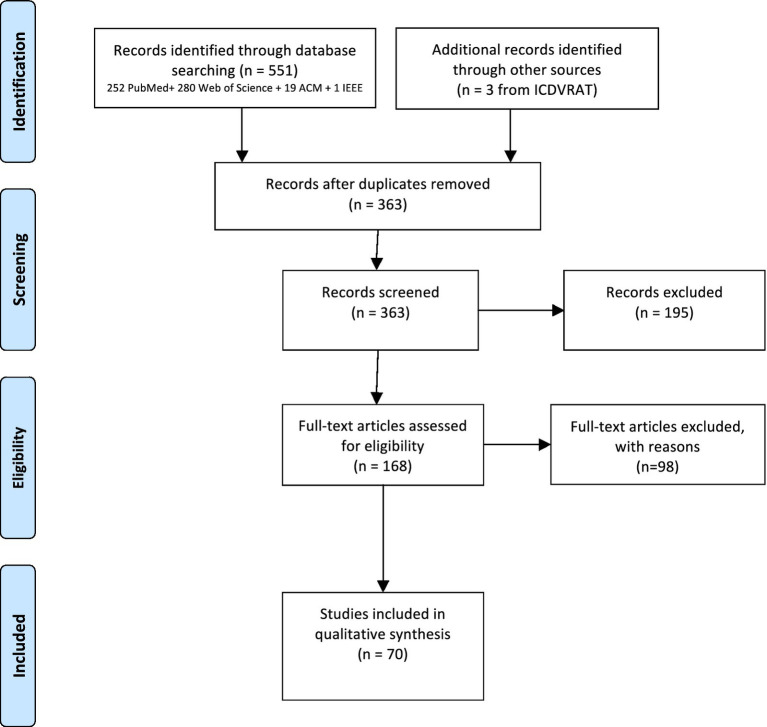
PRISMA flow chart.

The results of the different phases of the systematic review are depicted in the PRISMA flow diagram (Figure 1). A total of 551 papers were identified through database searching, 363 after duplicates removal. In the first screening based on titles and abstracts, 195 were removed mainly due to the type of study (review articles, theoretical articles, studies describing tools with no clinical validation). In total, 168 full-text articles were assessed for eligibility, being that 98 were excluded as they did not involve VR, did not include ABI participants, did not describe rehabilitation or assessment studies, or did not comprehend simulated environments or ADL. Accordingly, 70 articles were included for analysis in this systematic review. From the total of 70 studies, 45 had an experimental design and 25 a non-experimental design. Due to the reduced number of participants per trial and heterogeneity of outcome measures – 136 different cognitive, functional, motor, emotional, cyber sickness, immersion and engagement assessment instruments and questionnaires - it was not possible to perform a meta-analysis.

### 3.1. Kitchens

Simulation of kitchens is attractive for rehabilitation as it involves manipulation of objects, planning, execution of ADLs, and using skills that are commonly affected after ABI (Poncet et al., 2017) (Table 1). Twelve studies using kitchens were identified (six prospective cross-sectional studies, four performed case studies and two exploratory studies), which enrolled 132 stroke participants, 107 TBI participants, one participant with meningoencephalitis, and 54 healthy controls (HC) in activities performed in simulated virtual kitchens. Virtual kitchens were used to assess or rehabilitate executive functions (*n* = 4), attention (*n* = 4), upper limb function (*n* = 5), and engagement (*n* = 1), while performing different tasks such as preparing meals or hot drinks.

Most of these studies have focused on cognitive aspects involved in the performance of ADLs. For example, Zhang et al. (2001) used a kitchen environment with 30 participants with TBI to evaluate their ability to process and sequence information in comparison to healthy controls (HC). Individuals with TBI showed a decreased ability to process information, identify logical sequencing, and complete the overall task in comparison to HC (Zhang et al., 2001). A more complex meal preparation task that consisted on 81 steps was used in a later study of the same group. The scoring method and experimental setup were replicated but, in this case, a TV screen provided the visual feedback. The authors compared the performance of 54 participants with TBI in the virtual world and in a real physical kitchen and repeated the same procedure 3 weeks later. Correlation between scores in the virtual and in the real kitchen was moderate for both the first and second trial (Zhang et al., 2003).

Hilton et al., 2002 developed a tangible user interface (TUI) for a hot drink preparation task and analyzed the time to task completion and errors of seven stroke participants. The location of objects, the instructions given, the physical constraints, the ineffective user response, and the visual and auditory feedback were pointed as important aspects when recreating kitchens in VR. Moreover feedback was given for the TUI redesign (Hilton et al., 2002). Subsequently, Edmans et al. (2006) compared the performance of 50 individuals with stroke at preparing a hot drink task, consisting of 12–27 steps in a virtual and in a physical kitchen. The VE was displayed and interacted with a touch screen, and the performance was equally assessed as the number of steps that were completed. Weak correlations were found in the scores between conditions (*r* = 0.30, *p* < 0.005). Later on, these authors evaluated the effectiveness of this VE presented in a touch-sensitive screen in a sample of 13 single case studies with stroke patients. Visual inspection of scores across all cases showed a trend toward improvement over time in both real and virtual hot drink making ability in both control and intervention phases. There was no significant difference in the improvements in real and virtual hot drink making ability during all control and intervention phases in the 13 cases. The authors concluded that more development and studies on their system were required (Edmans et al., 2009). The hot drink preparation task in a virtual kitchen was also used by Besnard et al. (2016). The performance of 19 HC and 19 TBI was compared in both virtual and physical kitchen. Significant moderate correlations were found between the number of total errors in both environments for HC (*r* = 0.65, *p* = 0.001) and TBI (*r* = 0.44, *p* = 0.02). Measures of four virtual tasks significantly correlated with both executive and intelligence measures. Intelligence quotient correlated significantly with time to completion (*r* = 0.48, *p* = 0.03), total errors (r = 0.48, p = 0.03), and commission errors (r = 0.48, p = 0.03). The profile scores of the Zoo Map Test (Wilson et al., 1996) correlated significantly with total errors (*r* = 0.54, *p* = 0.01) and commission errors (*r* = 0.47, *p* = 0.03). In addition, a significant correlation was observed between the Modified Six Elements Test profile (MSET) score (Wilson et al., 1996) with the total number of errors (*r* = 0.60, *p* = 0.005) and omissions (*r* = 0.48, *p* = 0.03) (Besnard et al., 2016). Cao et al. (2010) also compared the hot drink preparation task in a physical kitchen and in a virtual replica, which was shown using a computer screen and interacted with a keyboard and a mouse. A study involving this VE and 13 HC and seven individuals post-stroke showed that participants with stroke needed nearly twice the time as HC, and made more errors in the VE than in the physical kitchen (Cao et al., 2010).

Virtual kitchens have also been used to assess and improve motor performance. Adams et al. (2015) simulated a meal preparation task consisting of 17 steps (Adams et al., 2015). The VE was shown in a TV screen and interacted using arm movements detected with the Xbox Kinect sensor. The time that 14 participants with stroke spent to complete the virtual task was moderately correlated (*r* = 0.56, *p* = 0.036) with their time score in the Wolf Motor Function Test (WMFT) (Wolf et al., 2001). Huang et al. (2017) used an Amadeo (Tyromotion GmbH, Graz, Austria), a hand rehabilitation robotic device, and an HMD, for upper-limb rehabilitation. Eight stroke participants were enrolled in a prospective cohort study for 18 30-min training sessions over 6 weeks. Each session consisted of passive (10 min), assist-as-needed (10 min), and two-dimensional and three-dimensional VR task-oriented exercises (5 min each) displayed in the HMD. Among the VR-based exercises, participants were required to complete certain ADLs on a simulated kitchen (i.e., open the oven, set the alarm clock) with the paretic hand. All the participants were assessed prior to and at the end of the intervention with the Fugl-Meyer Assessment (FMA) (Fugl-Meyer et al., 1975), the Motor Assessment Scale (MAS) (Malouin et al., 1994), and, in addition, with the active range of motion and force intensity of fingers collected by the robotic device. Results showed an improvement in motor skills of all eight subjects, which was evidenced by an increase of 2.32 (+37.5%) in the FMA, 1.16 (+38.8%) in the MAS, and an increase of 3.36 (+42.8%) and 11.21 (+33.3%) in the average mean force during extension and flexion, respectively. All participants but one also showed noticeable improvement in range of motion (Huang et al., 2017).

Triandafilou et al. (2018) have developed the Virtual Environment for Rehabilitative Gaming Exercises (VERGE) system for home motor therapy purposes. Within this 3D multi-user VE, stroke patients could interact with therapists and/or other stroke patients. Each user’s own movement controls an avatar through kinematic measurements made with a Kinect device. The system (laptop, Xbox Kinect Sensor, and a Wireless Optical Pen Mouse) was designed to train important movements for rehabilitation and to provide real-time feedback of performance. The VERGE system includes 3 tasks: (1) Ball Bump, where the goal is to hit a ball back and forth across the table, while avoiding objects on a table; (2) Food Fight, where the user “grasps” an object by placing the avatar’s hand in close proximity and clicking a button on a wireless optical pen mouse with either hand; and (3) Trajectory Trace, where the participant draws a 3D trajectory in the air. This trajectory is then passed to another participant who attempts to erase it by retracing. The state of the game (Draw, Claim, Trace, or Reset), as well as the initiation and termination of drawing the curve, is controlled by touching a button (located on the avatar’s chest) with the less affected hand. Fifteen stroke patients with upper extremity hemiparesis participated in a pilot study, consisting of a three-week intervention. For each week, the participant performed three 1-h training sessions with one of three modalities: (1) VERGE system, (2) an existing VE based on Alice in Wonderland (AWVR), or (3) a home exercise program (HEP). Participant kinematics was captured with the Xsens 3D motion tracker system (MVN, Xsens, Culver City). Arm displacement averaged 350 m for each VERGE training session. Arm displacement was not significantly less when using VERGE than when using AWVR or HEP. Participants were split on preference for VERGE, AWVR or HEP. The VERGE system was found to be an effective means of promoting repetitive practice of arm movement by 85% of the participants and almost all of them indicated a willingness to perform the training for at least 2–3 days per week at home (Triandafilou et al., 2018). Finally, Thielbar et al. (2020) evaluated the VERGE performance with stroke patients during longitudinal studies in a laboratory environment and in participants’ homes. Active arm movement was tracked throughout therapy sessions for both studies. In the end, patients achieved considerable arm movement while using the system. Mean voluntary hand displacement was greater than 350 m per therapy session for the VERGE system. Compliance for home-based therapy was high since 94% of all scheduled sessions were completed. Having multiple players led to longer sessions and more arm movement than when the patients trained by themselves, corroborating the benefits of this multiuser VR system (Thielbar et al., 2020).

It is important to highlight that familiarity with the environment may have an impact on the level of engagement. O’Brien (2007) replicated the kitchen of three individuals with stroke in VR. The VE was shown using an HMD and navigation was facilitated indicating the direction to the investigator through basic hand gestures using their affected limb. The level of engagement of the participants while navigating the virtual version of their kitchen or an unfamiliar one was assessed using a semi-structured interview, questionnaires, and variations in skin conductance. Higher levels of engagement were associated to greater familiarity with the environment (O’Brien, 2007).

### 3.2. Supermarkets

Supermarkets have also been repeatedly simulated in VR as shopping involves planning and execution skills, which are also likely affected after an injury to the brain (Josman et al., 2006; Kang et al., 2008; Raspelli et al., 2012; Yip and Man, 2013; Sorita et al., 2014; Yin et al., 2014; Cogné et al., 2018; Ogourtsova et al., 2018; Demers and Levin, 2020; Spreij et al., 2020b; Table 2). Shopping is a high-demanding activity with strong association to independence, which is difficult to assess in real-life environments and difficult to replicate in the clinical setting (Maggio et al., 2019). These factors have motivated the use of virtual supermarkets to assess and train individuals with cognitive disorders. The search resulted in 11 studies, two non-experimental and nine experimental, involving 312 participants with stroke, 33 participants with TBI, 37 participants with ABI of unspecified aetiology, and 134 HC. Virtual supermarkets have mainly focused on executive functions (*n* = 7), but also on memory (*n* = 3), attention (*n* = 1), upper-limb motor function (*n* = 2), and neglect (*n* = 1).

From the 11 studies, four involved the Virtual Action Planning Supermarket (VAPS), a virtual supermarket where users are required to purchase seven items from a list of products. In the first study, Josman et al. (2006) examined the feasibility of using VAPS to assess and treat executive functions deficits of 26 individuals with stroke. The distance traveled, number of purchased items, correct and incorrect actions, number of pauses, and the time required to pay was used to assess performance in the virtual task. The VE was shown on a screen and participants used a keyboard and a mouse to navigate and select objects. Participants’ executive functions were assessed with the Behavioral Assessment of Dysexecutive Syndrome (BADS) (Wilson et al., 1996). Moderate correlations between virtual and clinical measures were found between the number of items purchased, mean correct actions, mean duration of the pauses, and the key search subtest from the BADS (*r* = 0.40, *r* = 0.47, and *r* = −0.44, *p* < 0.05, respectively) (Josman et al., 2006). In a later study, Sorita et al. (2014) studied the VAPS with 12 stroke, 33 TBI, and 5 with unspecified ABI aetiology participants. Performance in the VAPS and neuropsychological functioning (episodic memory, prospective memory, working memory index, perceptual organization index, processing speed, go/no-go errors, divided attention omissions, visual scanning omissions) was assessed and they also collected data from the Community Integration Questionnaire (CIQ) (Willer et al., 1993). A principal component analysis raised a 4-factor model that accounted for 70% of the total variance. Authors speculated that performance in the VAPS could be partially associated to neuropsychological processes, as measured by the assessment tools. Results pointed out that performance in the virtual supermarket could not be explained by executive functions alone, but it may have involved other cognitive processes, such as episodic and prospective memory, divided attention, and visual scanning (Sorita et al., 2014). In a third study, Josman et al. (2014) compared the performance of 24 stroke participants to that of 24 HC in the VAPS and the BADS test. Stroke participants were also assessed with the Revised Observed Tasks of Daily Living (OTDL-R) (Diehl et al., 2005), a performance-based test of everyday problem solving. VAPS was correlated with the BADS in the experimental group. Interestingly, the number of items purchased and the number of correct actions significantly correlated with the OTDL-R (*r* = 0.64, *p* = 0.001, and *r* = 0.68, *p* = 0.0001, respectively). In the latest study, Cogné et al. (2018) used VAPS to investigate how high-load non-contextual auditory stimuli affects navigational performance in stroke patients and its correlation with dysexecutive disorders. Four kinds of stimuli were considered: sounds from living beings, sounds from supermarket objects, beeping sounds and names of other products that were not available in the VAPS. The control condition did not have auditory stimuli. To assess executive functioning they used the Groupe de réflexion pour l’évaluation des fonctions exécutives (GREFEX) battery. The 40 stroke patients navigational performance decreased under the 4 conditions with non-contextual auditory stimuli, especially for those with dysexecutive disorders. Lower performance was related with more GREFEX tests failed in the 5 conditions. Patients felt significantly disadvantaged by the non-contextual sounds from living beings, supermarket objects and names of other products as compared with beeping sounds. Also, patients’ recall of the collected objects was significantly lower under the condition of names of other products. Moreover, left and right brain-damaged patients did not differ in navigational performance in the VAP-S under the five auditory conditions. One of the most important outcomes of this study was that non-contextual auditory stimuli could be used in neurorehabilitation paradigms to train patients with dysexecutive disorders to inhibit disruptive stimuli (Cogné et al., 2018).

Kang et al. (2008) simulated a virtual shopping task in another virtual supermarket that consisted of four aisles and four glass fronted fridges with 50 different items. The VE was displayed in an HMD and a joystick enabled navigation and item selection. The experimental sessions with the system consisted of three sub-tasks: (1) find three displayed items (assessement the interface and the visuospatial functions: visual attention, visuomotor coordination, and visual organization); (2) select the highlighted items with auditory or visual cues, and respond to unexpected events such as item dropping (assessment of immediate and delayed recognition memory, auditory and visual memory, and attention); and (3) select a shopping item according a given request and a designated price (assessment of planning, problem solving, and calculation). Authors compared the performance of 20 stroke participants with that of 20 HC and found significant differences between groups in the performance index, interaction error, delayed recognition memory score, auditory memory score, visual memory score, attention index, attention reaction time, and executive index (Kang et al., 2008).

In another study, Raspelli et al. (2012) evaluated a virtual version of the Multiple Errands Test (Alderman et al., 2003), the VMET, which was designed in a cost-free VR platform based on open-source software. The VE, consisting in a virtual supermarket with several shelves that displayed different items, was presented on a screen and interaction was facilitated with a joypad. Three groups of participants were included in the study and were required to select and buy several products in the VMET: nine individuals post-stroke, 10 young HC, and 10 older HC. Results showed moderate to strong correlations between performance in the VMET and the Test of Attentional Performance (Zimmerman and Fimm, 1992) for participants with stroke. Specifically, correlations were found between the time to complete the task in the VMET and the subtest for the state of alert with warning sign (*r* = 0.762, *p* = 0.028), total errors in the VMET with the subtest of incompatibility (*r* = 0.75, *p* = 0.019), and inefficiencies in the VMET with the subtest of attention shift with valid stimulus (*r* = 0.67, *p* = 0.045). Significant differences emerged among the three groups in the VMET. In general, young adults performed better than elderly adults, and both young and older HC participants performed better than individuals with stroke (Raspelli et al., 2012).

Yip and Man (2013) developed a virtual supermarket to be presented on a screen and interacted with a joystick or keyboard, according to each participant preference. The system consists of different tasks, such as buying discounted items, memorizing and picking items from a list, and discriminating those items that have a special tag from the rest, which allows to train prospective and retrospective memory, and inhibition, respectively. A total of 37 individuals with ABI of unspecified aetiology participated in a 5 to 6-week RCT. Participants were divided into an experimental group (*n* = 19), which trained prospective and retrospective memory and inhibition in the virtual supermarket, and a control group (*n* = 18), which participated in reading activities and table games. Prospective memory was assessed before and after intervention with the Cambridge Prospective Memory Test (CAMPROMT) (Wilson et al., 2005). Further assessments included the Hong Kong List Learning Test (Chan and Kwok, 2009), the Frontal Assessment Battery (FAB) (Dubois et al., 2000), the Word Fluency Test (Marsh and Hicks, 1998; Henry and Crawford, 2004), the Community Integration Questionnaire (Willer et al., 1994), a customized self-efficacy questionnaire, and a battery of tasks in a real convenience store. Participants had to perform three event-based tasks (calling back home when participants saw particular products or writing down the price of particular drinks), two time-based tasks, and had to shop for eight items according to a shopping list. The experimental group, in addition, completed a VR-based test that consisted of three event-based and three time-based tasks with a difficulty comparable to the most difficult level in the VR program. Results showed moderate correlations between the real life event-based tasks and most of the VR-based measures, including accuracy of event-based (*r* = 0.58, *p* < 0.01), time-based (*r* = 0.48, *p* < 0.05), and on-going tasks performance (*r* = 0.52, *p* < 0.05). The accuracy of time-based tasks in both VR and real life were also moderately correlated (*r* = 0.55, *p* < 0.05). After the intervention, significant improvements were found in the experimental group in most VR-based measures, such as, immediate recall, on-going tasks performance, and number of time checks; and in event-based (*p* < 0.01) and time-based tasks (*p* < 0.01) of the real-life test. No significant difference was found in any outcome measure for the control group (Yip and Man, 2013).

Virtual supermarkets have also been used to train upper-limb function. Yin et al. (2014) used a virtual supermarket where participants had to pick a virtual fruit from a shelf and release it into a virtual basket as many times as possible within a two-minute trial. The VE recreated a local supermarket aiming to increase familiarity and engagement. Interaction was facilitated by a Sixense hand-held remote controller (Sixense Entertainment, USA) in such a way that participants held the controller with their affected hand, and their movements were detected and transferred to a virtual hand avatar in the VE. In a RCT with this simulated supermarket, participants were randomly divided into an experimental group (*n* = 11) and a control group (*n* = 12). The experimental group received nine 30-min VR-based training sessions plus additional physical and occupational therapy. The control group received time-matched physical and occupational therapy. Assessment of motor function included the FMA (Fugl-Meyer et al., 1975), the Action Research Arm Test (ARAT) (Lyle, 1981), the Motor Activity Log (Taub et al., 1993) and the Functional Independence Measure (FIM) (Grimby et al., 1996) and was conducted at baseline, post intervention, and 1 month post-intervention. Although both groups improved their performance, results showed non-significant differences in all clinical measures either from baseline to post-intervention, or to follow-up (Yin et al., 2014). Recently, Demers and Levin (2020) explored if reach-to-grasp movements in a low-cost 2D VE were kinematically similar to those made in a physical environment (PE) in healthy controls and stroke patients. In their study, participants (HC = 15, Stroke = 22) had to make unilateral and bilateral reach-to-grasp movements in a 2D VE and a similar PE. The VE was a grocery-shopping task with two scenes representing aisles filled with typical supermarket items presented in a large screen and interacted through a Kinect II camera. The hands and forearms were represented by avatars viewed from a first-person perspective. The PE included only the object to be grasped since additional objects in would have interfered with the ability of the camera to track the arm movement. Temporal and spatial characteristics of the endpoint trajectory, arm and trunk movement patterns were compared between environments and groups. Hand positioning at object contact time and trunk displacement were unaffected by the environment. Compared to PE, in VE, unilateral movements were less smooth and time to peak velocity was prolonged. In HC, bilateral movements were simultaneous and symmetrical in both environments. In stroke, movements were less symmetrical in VE. Authors considered that using a low-cost 2D VE might be a valid approach for upper-limbs rehabilitation after stroke (Demers and Levin, 2020).

Ogourtsova et al. (2018) examined the feasibility of a functional shopping activity, the Ecological VR-based Evaluation of Neglect Symptoms (EVENS), in the assessment of USN. The EVENS consists of simple and complex 3D scenes depicting grocery-shopping shelves, where joystick-based object detection and navigation tasks are performed while seated. The authors compared the effects of virtual scene complexity on navigational and detection abilities in right hemisphere stroke patients with USN (*n* = 12) and without USN (*n* = 15) and in age-matched HC (*n* = 9). Participants with USN demonstrated longer detection times, larger mediolateral deviations from ideal paths and longer navigation times in comparison to participants without USN and HC. The EVENS detected lateralized and nonlateralized USN related deficits, performance alterations that were dependent or independent of USN severity, and performance alterations in 3 USN participants compared to HC (Ogourtsova et al., 2018).

In a different perspective, Spreij et al. (2020b) examined the feasibility and user-experience of a virtual supermarket by comparing non-immersive VR through a computer monitor and immersive VR trough a HMD. The virtual supermarket was modeled according to a regular Dutch supermarket with 18 shelves, eight cash registers, 20.000 real brands products (e.g., bread, fruit, vegetables) and freezing compartments. The VR task consisted in finding three products from a shopping list, and passing through the cash registers. Two different grocery lists were randomly presented over three trials (15 min each), and participants were asked to recall the products. In total, 88 stroke patients and 66 HC performed the VR task twice, with the computer monitor (a wired Xbox 360© controller was used to navigate) and with the HMD (Oculus Rift DK2© with an Xbox 360© or the HTC Vive© with its own controllers), being these conditions randomized. Although both stroke patients and HC reported an enhanced feeling of engagement, transportation, flow, and presence when tested with a HMD, more negative side effects were experienced, especially with the older Oculus Rift DK2. Most stroke patients had no preference for one interface over the other, yet younger patients tended to prefer the HMD. The HMD seems preferable in neuropsychological assessment since it induces more natural behavior, but a computer monitor remains a valid alternative (Spreij et al., 2020b).

### 3.3. Shopping malls

In addition to the required skills to cope with supermarkets, shopping malls require navigation and search for specific locations in more open spaces. The complexity of the distribution of shops and their interconnecting walkways, and the sensory overload can pose a challenge for individuals with attention deficits, as those commonly experienced after an injury to the brain, and consequently affect cognitive and motor performance (Koch et al., 2018). These factors have motivated the creation of virtual shopping malls for assessment and/or rehabilitation of cognitive and/or motor functions (Table 3). Ten studies have been included in this analysis, which involved 62 individuals with stroke, 67 individuals with TBI, and 233 healthy individuals, in four validation studies, three pilot studies, two evaluation studies, and a single-subject design. Shopping malls have been used to assess or train attention (*n* = 1), executive functions (*n* = 9), memory (*n* = 2) and upper limb function (*n* = 2). Eight of the ten studies used the same VE, the VMall, or its evolution, the VIS (Rand et al., 2007, 2009a,b,c; Hadad et al., 2012; Erez et al., 2013; Jacoby et al., 2013; Nir-Hadad et al., 2015). The remaining two studies used a touch panel or a keyboard and a mobile phone to interact with the VE (Okahashi et al., 2013; Canty et al., 2014).

The VMall simulates a large mall where users can navigate through aisles. The VE runs on the IREX® platform (GestureTek, Toronto, Canada) and is interfaced by sustained arm reaches, which are captured through color tracking. The VMall engage participants in a complex everyday shopping task that trains upper extremities and executive functions, primarily planning, multitasking, and problem solving. Rand and colleagues investigated the potential of VMall as an evaluation tool in a study that compared the performance of 14 post-stroke participants to that of 93 HC (Rand et al., 2007). All participants were required to shop four grocery items in the VMall and complete the Short Feedback Questionnaire (SFQ) (Witmer and Singer, 1998) and the Borg’s Scale of Perceived Exertion (Borg, 1990). Total shopping time was, in general, significantly longer for participants with stroke, possibly due to their overall impaired motor control and EF. Significant differences were found for the Borg’s Scale of Perceived Exertion between the groups [*F*(3,61) = 5.9, *p* < 0.001] while no significant differences emerged in the SFQ. A later study by the same group investigated the effectiveness of an in-home intervention with the VMall in individuals with stroke (Rand et al., 2009a). Six stroke participants received ten 1-h treatment sessions over a period of 3 weeks and the motor and functional abilities of their weaker upper extremity were assessed before and after the intervention. Results showed a relative improvement in the FMA (0.24 ± 0.24), the ability score of the Wolf Motor Function Test (WMFT) (0.30 ± 0.34), and in the numbers of tasks performed using both hands of the Questionnaire of Upper Extremity Function in Daily Life (Broeks et al., 1999) (0.22 ± 0.4). In a later study, authors explored the effectiveness of an intervention with the VMall to improve multitasking deficits after stroke (Rand et al., 2009c). Four participants received ten 60-min sessions with the system over 3 weeks. Assessment included the Multiple Errands Test in both a real mall and the VMall. Participants showed improvements in most of the error-based measures (total number of mistakes, rule break mistakes, inefficiency mistakes, use of strategies mistakes) that ranged from 20.5 to 51.2%. The ecological validity of the VMall was analyzed in a later study by the same group (Rand et al., 2009b). Authors compared the performance of three groups of participants (stroke, young and older HC participants) in an adaptation of the Multiple Errands Test that was administered in a real shopping mall and in the VMall. The executive function of stroke participants was assessed by the Zoo Map Test of the BADS and their level of independence during instrumented ADLs by an *ad-hoc* questionnaire. Significant moderate to high correlations were found between the performance in the real and virtual scenario for both post-stroke participants [total number of mistakes (*r* = 0.70, *p* = 0.036), partial mistakes (*r* = 0.88, *p* = 0.002), inefficiency mistakes (*r* = 0.73, *p* = 0.025)] and older healthy participants [total number of mistakes (*r* = 0.66, *p* = 0.01), complete mistakes (*r* = 0.58, *p* = 0.01), partial mistakes (*r* = 0.61, *p* = 0.01), and inefficiency mistakes (*r* = 0.66, *p* = 0.01)]. The virtual version of the test was able to differentiate between younger and older healthy participants, and also between healthy and stroke participants. Concerning stroke participants, significant moderate to high correlations were found between the Zoo Map test and performance in both the real and virtual version of the Multiple Errands Test. Specifically, the Zoo Map Test correlated with the number of errors (*r* = −0.93, *p* < 0.000), partial mistakes in completing tasks (*r* = 0.80, *p* < 0.009), non-efficiency mistakes (*r* = 0.86, *p* < 0.003), and the time to complete the Multiple Errands Test (*r* = 0.79, *p* < 0.012) and also, with the non-efficiency mistakes in the virtual version of the test (*r* = −0.76, *p* < 0.04). Also for participants with stroke, significant moderate to high correlations were found between scores in the questionnaire and rule breaks in the Multiple Errands Test (*r* = 0.80, *p* < 0.03), and mistakes in both the real (*r* = −0.76, *p* < 0.04), and the virtual version of the test (*r* = −0.82, *p* < 0.02). In another study, Jacoby and colleagues performed a pilot RCT to examine the effectiveness of the VMall at improving EF after TBI (Jacoby et al., 2013). Twelve participants were randomized either into an experimental group, who trained planning and execution of shopping tasks with restricted budget in the VMall, or a control group, who participated in a conventional occupational therapy program. All the participants received ten 45-min and were assessed before and after the intervention with the Multiple Errands Test and the Executive Function Performance Test (Baum et al., 2009). No significant differences between groups emerged before and after intervention. However, participants in the experimental group showed greater relative improvement in comparison to the control group in both outcomes (*z* = −1.761, *p* = 0.046; and *z* = −1.761, *p* = 0.046, respectively). Finally, the VMall was also used in a study that compared the performance of 20 children with TBI in a simple virtual shopping task with that of 20 typically developing peers (Erez et al., 2013) to determine the feasibility of the VE in pediatric population with TBI. Participants were required to shop four items in the VMall and their planning abilities were evaluated with the Zoo Map subtest of the BADS test for children. Different performance between groups was detected in the mean shopping time (z = −3.05, *p* = 0.002) and number of mistakes (z = −1.95, *p* = 0.068), which were significantly higher for children with TBI. In addition, time to complete the shopping task in the VMall and the Zoo Map score correctly classified 75% of the participants into each group, whereas time to complete the shopping task alone correctly classified 65% of the participants.

Two studies included in this review used the newer version of the VMall, the VIS, which, unlike its predecessor, allows for creating different shopping malls by changing and customizing the stores, and types, quantities, and locations of the groceries in each store. The VE runs on the SeeMe system (Brontes Processing, Poland), and is similarly interfaced by sustained arm reaches, but detected with the Microsoft Kinect. Navigation within and between shopping aisles and selection of groceries is facilitated by virtually “touching” directional arrows and “hovering” over photos of the groceries, respectively. Nir-Hadad et al. (2015) compared the shopping performance of stroke and healthy participants in three different environments: a real environment (hospital cafeteria), a store mock-up (physical simulation), and the VIS. Five stroke individuals and six HC were required to purchase four grocery items in the VIS and the store mock-up, and to repeat the task with budget constraints in all three environments. Results showed that post-stroke participants required more time to finish the task than HC in all the environments. In addition, for both groups, time to complete the task within the VIS was longer than that in the store mock-up and the cafeteria (Hadad et al., 2012). A later study by the same authors examined the discriminant, construct-convergent, and ecological validity of the purchasing task for assessing the performance in the instrumental ADLs. A supermarket, a toy store, and a hardware store were recreated in the VIS, and 19 people with stroke and 20 HC performed the shopping task in the VE and a real shopping environment (the same hospital cafeteria of the previous study) in counterbalanced order. Executive functions of all the participants were also assessed, among other tests, with the Rule Shift Cards subtests from the BADS (Wilson et al., 1996), and the Telephone Use and Bill Payment subtests of the Executive Function Performance Test. Concerning the discriminant validity, the control group significantly required less time to complete the shopping task (*U* = 55.50, *p* = 0.001) and traveled shorter distance (*U* = 81.00, *p* = 0.002) in the VIS than participants with stroke. Best performance was detected in HC in the cafeteria, evidenced by significantly fewer budget excesses (*U* = 111.00, *p* = 0.007) and assistance from the cashier (*U* = 96.50, *p* = 0.008). Concerning convergent and ecological validity, significant moderate correlations emerged between the performance of both the control group [time to the first purchase (*r* = −0.49, *p* < 0.05), total time (*r* = −0.47, *p* < 0.05), and number of errors (*r* = −0.46, *p* < 0.05) and the stroke group (distance traveled in VIS and time to the first purchase in the cafeteria: *r* = 0.61, *p* < 0.01)]. Better performance in the clinical assessments of EF was related to better performance in the VIS for all participants. For HC, the Telephone Use significantly correlated with the time of total purchase in the VIS (*r* = 0.51, *p* < 0.05), and the Rule Shift Cards significantly correlated with the distance traversed in the VIS (*r* = −0.57, *p* < 0.01). For stroke participants, significant correlations emerged between the Bill Payment and the time until the first purchase (*r* = 0.57, *p* < 0.05) and for total purchase in the VIS (*r* = 0.56, *p* < 0.05), and also between the Telephone Use and the time for total purchase in the VIS (*r* = 0.55, *p* < 0.05) and between the Rule Shift Cards and the time until the first purchase in the VIS (*r* = −0.53, *p* < 0.05) (Nir-Hadad et al., 2015).

Okahashi and colleagues developed the VST, a virtual Japanese-style shopping mall, to assess general cognitive function (Okahashi et al., 2013). In the virtual test participants are required to memorize items to buy, look for specific shops on a street, choose items in a shop, and perform different tasks. The VE is presented a multi-touch display and interacted through finger touches. The convergent validity of the VST with conventional tests [Mini-Mental State Examination (MMSE) (Folstein et al., 1975), Symbol Digit Modalities Test (Smith, 1982), Simple Reaction Time Task (Beck et al., 1956), Rivermead Behavioral Memory Test (Wilson et al., 1987a), Everyday Memory Checklist (Kazui et al., 2006)] and the effect of age were investigated in a study that involved five stroke and five TBI participants and ten age-matched HC. Results evidenced moderate to high correlations between measures of the VST and conventional tests. Regarding attention, total time in the VIS correlated with the completing rate of the Symbol Digit Modalities Test (*r* = −0.80, *p* < 0.01) and the correct rate of the Simple Reaction Time Task (*r* = −0.89, *p* < 0.01). Regarding memory, bag use in the VST correlated with the pictures score of the Rivermead Behavioral Memory Test (RBMT) (*r* = −0.65, *p* < 0.05), list use correlated with the standard profile score (*r* = −0.71, *p* < 0.05), the belonging score (*r* = −0.67, *p* < 0.05), and appointment score of the of the RBMT (*r* = −0.73, *p* < 0.05), number of correct purchases correlated with date score of the RBMT (*r* = 0.67, *p* < 0.05), and total time correlated with the standard profile score (*r* = −0.71, *p* < 0.05), and the appointment score of the RBMT (*r* = −0.88, *p* < 0.01). In addition, participants with brain damage required more hints (*p* < 0.05) and made more movements (*p* < 0.05) to perform the task than healthy controls. Older healthy participants significantly spent more time to perform the task than younger HC (*p* < 0.01).

Another virtual mall, the Virtual Reality Shopping Task (VRST), was presented by Canty and colleagues (Canty et al., 2014). The VE simulated a shopping center where you can navigate through different stores and interact with a mobile phone, a stores map, and a list of tasks. The VE is presented in a TV screen and interacted with a keyboard. The authors evaluated the sensitivity of three VR-based shopping tasks, their ecological validity, and their convergent validity with the Lexical Decision Prospective Memory Task (LDPMT) (Einstein and McDaniel, 1990). Thirty individuals with severe TBI and 24 HC were required to purchase items in a pre-specified order in a selection of shops, texting in a virtual mobile phone at different moments, and pressing a key when a sale announcement was heard. Results showed that the performance of individuals with TBI was significantly worse than that of controls on time and event-based tests in both the VRST and the LDPMT. Results of the participants with TBI showed moderate correlations between event-based components and performance on the prospective memory tasks of the VRST with the LDPMT (*r* = −0.657, *p* < 0.001) and with the total prospective memory performance on the VRST and event-based performance on the LDPMT (r = 0.662 p < 0.001) (Canty et al., 2014).

### 3.4. Streets

Sixteen studies have been included in this review that involved 418 individuals with stroke, 90 individuals with TBI, 6 ABI individuals with unspecified etiology, six individuals with other type of brain injury including cerebral tumor and cortical cyst and 102 healthy participants. Streets have been recreated in VR to assess or train Unilateral Spatial Neglect (USN) (*n* = 6), driving skills (*n* = 4), and route retraining (*n* = 4) (Table 4).

Safe street crossing is specially demanding for individuals with hemispatial neglect, as it requires dealing with cars coming from both sides. Three studies included in this review used the City Street. The VE consists on typical local city street with an avatar facing a crosswalk and vehicles approaching from different directions at different speed, and allows for practicing street crossing with different levels of difficulty. The VE was shown on a 15-inch PC monitor or projected on a wall, and the interaction facilitated with a keyboard. In a first study, Naveh et al. (2000) carried out a feasibility study comparing the performance of six individuals with stroke (four with USN) and six HC in the City Street. Participants performed a number of sessions with the VE that varied from one to four and had different durations (from 30 to 60 min). In each session, participants practiced street crossing with progressive difficulty as they completed the previous levels of difficulty. Although no statistical differences were examined, participants with stroke seemed to require more time to complete the task (Naveh et al., 2000). A later study by the same group, with the same procedure and number of participants, reported that HC took less time to complete the task, looked less frequently at oncoming traffic, and had fewer accidents than patients when the difficulty increased. As in the previous study, no statistical analysis was performed. In this latter study, the authors also examined the effect of display size on subject performance in the virtual task and found that, although participants looked to both sides more often when the VE was displayed on a projection, they had more accidents and needed more time to complete the tasks (Weiss et al., 2003). Again in a later study, the same group evaluated the effectiveness of City Street in a sample of post-stroke USN participants. Participants were pseudo-randomized to a control group (*n* = 8), who trained with a computer-based visual scanning task, or to an experimental group (*n* = 11), who trained street crossing in the City Street. Intervention in both groups consisted on twelve 45-min sessions administered three times a week. Participants were assessed before and after the intervention, with a battery of tests that evaluated the level of neglect [Star Cancellation Test of the Behavior Inattention Test (BIT) (Wilson et al., 1987b) and the Mesulam Cancelation Test (Mesulam, 2000)], and performance during street crossing in the VE (number of times that participants looked to the left and number of accidents) and in the real world (number of times that participants looked to the left, and decision time). Performance in the real world was assessed from videotaped recordings. Although performance of both groups seemed to improve after the intervention, no remarkable differences appeared between groups (Katz et al., 2005).

Navarro et al. (2013) also developed a VR system to practice street-crossing, which consisted of a 47-inch LCD screen that displayed the VE, an infrared tracking system that detected head turns, and a joystick that enabled navigation in the virtual world. The VE is shown from a first-person perspective and consists on a crosswalk that intersects two two-way roads with median strips with traffic approaching simulating real-life. Participants were required to safely navigate from an origin point to an end at the other side of the road and come back. A total of 15 HC and 32 stroke participants (17 with USN and 15 with no USN) were included in a validation study. Authors found that HC had better performance in terms of time to complete the task and safety. Similar tendency emerged when considering participants with stroke: participants without USN finished the task faster (*F* = 28.9, *p* < 0.01) and more safely (*F* = 55.8, *p* < 0.01) than those with USN. Importantly, the presence of neglect was a significant predictor of the number of accidents (*t* = 6.5; *p* < 0.001). Regarding convergent validity, the time to complete the virtual task and the number of accidents had moderate to strong correlations with timed tests, such as the Conner’s Continuous Performance Test (Conners et al., 2003) (*r* = 0.5–0.6, *p* < 0.05) and the Color Trail Test (CTT) (D’Elia et al., 1994) (*r* = 0.55–0.75, *p* < 0.05). In addition, the score in the BIT showed moderate to strong correlations with the number of accidents (*r* = −0.77, *p* < 0.01) and the number of head turns to the left (*r* = 0.4, *p* < 0.05) (Navarro et al., 2013).

Kim et al. (2007) have also proposed a HMD system to train stroke patients with USN for street crossing. The training procedure consisted of completing missions while keeping the virtual avatar safe when crossing the street. While searching for a virtual vehicle appearing on the right or left side of an avatar, the subject had to respond to the system by pushing the mouse button when he found the car. When the subject did not recognize the car’s movement at the two-third distance from a starting position to the avatar position, the car turned on headlights (visual cue) to notify the subject that the car was approaching an avatar. Despite the visual cue, if he could not recognize the car’s movement, the car would give him an alarm sound at one-third of the distance from a starting position to the avatar position. The system’s difficulty was controlled by level (car velocity) and stage (distance between an avatar and a subject). The authors compared the performance of 10 stroke patients with USN with the performance of 40 HC considering the following parameters: deviation angle, reaction time, right and left reaction time, visual cue, auditory cue and failure rate of mission. Additionally, traditional methods such as line bisection and cancellation tests were analyzed. Results showed that the proposed VE system was proper to USN training and had an effect in the stroke group (*r* = 0.81) (Kim et al., 2007).

Finally, for virtual road crossing assessment, Wagner et al. (2021) developed the iVRoad to detect discrete symptoms of USN in right-hemispheric post-stroke patients. The task consisted of dropping a letter in a mailbox on the way to work and was presented through an HTC Vive. To do so, the users first had to safely cross two roads and the square in between, and then return to the starting position to continue his/her way to work. The authors performed a study with 18 stroke patients to evaluate iVRoad with respect to usability, satisfaction, sense of presence and cybersickness. Moreover, they examined patients with and without USN and identified parameters for distinguishing patients with and without USN, such as the decision time, the error rate and the head direction ratio. The interaction with iVRoad through the HTC Vive Controller could be used without difficulties by all patients (Wagner et al., 2021).

Driving simulators have been used in three studies to examine the driving skills of persons with ABI (Wald et al., 2000; Akinwuntan et al., 2005; Devos et al., 2009; Wagner et al., 2021). Wald et al. (2000) presented the DriVR, a VR driving simulator that consisted of ten testing scenarios presented in an HMD, which appear in a continuous sequence as the participant drives through a small town roughly 1.4 km square. The authors compared indicators of driving ability of 28 adults with TBI in the DriVR with their performance in an on-road test and in a video test, the Driver Performance Test II, and with their performance in the Trail Making Test (TMT) (Reitan, 1958) and Adult Visual Perception Test (Baylor University Medical Center, n.d.) (Wald et al., 2000). Performance in the VR test did not show any correlation with the on-road and video driving tests, and only reached moderate correlations for the ability to maintain the lane while passing parked cars and as oncoming cars pass in the DriVR with the number of fails in the on-road test (r = 0.56 and r = 0.50, respectively). Poor correlations with no statistical significance were found between performance in the DriVR and in the neuropsychological assessment tests. In another study, the same group explored the effect of a simulator-based training on driving after stroke. The simulator consisted of a full-bodied car with all its original mechanical parts and the VE was projected on a screen of size 2.3×1.7 m and a visual angle of 45°. The VE represented an interactive 13.5 km scenario. A total of 73 stroke individuals participated in this study and were randomly allocated in to either an experimental (simulator-based training) or control (driving-related cognitive tasks) group. Both groups completed a total of 15 one-hour sessions administered three times a week and were assessed before and after the intervention with an on-road test and with the Stroke Driver Screening Assessment, and 6 months after the intervention with an official pre-driving exam. Although both groups improved after the training, no significant differences were detected between groups in any measure but in the sign recognition. However, a greater number of participants in the experimental group improved their classification in the on-road test, even though the difference did not reach statistical significance. More importantly, 73% of the participants in the experimental group were legally allowed to drive, in contrast to 42% of those in control group (Akinwuntan et al., 2005). A later item-per-item analysis of the data showed that the training in the simulator provided greater improvements in visual behavior, perception and anticipation of the road signs and the traffic, and turning left (Devos et al., 2009).

Virtual streets have also been used to assess and rehabilitate skills related to route retraining (Titov and Knight, 2005; Lloyd et al., 2006, 2009; Sorita et al., 2013). Titov and Knight (2005) developed a system to simulate a street scene using photographs and sounds that are displayed and interacted on a touch screen to assess the ability to remember instructions. Two individuals with stroke and an individual with TBI completed three tasks in the virtual street. In the first task, participants had to remember five errands. In the second and third tests, participants had to follow instructions; the former had a list with the items to facilitate the task, but not the latter. Participants were also assessed with a battery of neuropsychological tests that included Digit Span and Word Lists sub-tests from the Wechsler Memory Scale-III (WMS-III) (Wechsler, 1997a), the National Adult Reading Test (Nelson and Willison, 1991), the Wechsler Adult Intelligence Scale-III (WAIS-III) (Wechsler, 1997b), the Wisconsin Card Sorting Test (WCST) (Heaton, 2005), FAS Test (Benton, 1968), and the Stroop Test (Spreen and Strauss, 1991). Performance of individuals with brain injury in the VE was compared to that of three-matched HC, and showed better performance of the HC (Titov and Knight, 2005).

Lloyd et al. (2006, 2009) used an off-the-shelf gaming console, the Playstation 2 (Sony, Tokyo, Japan) and a driving videogame, the Driv3r (Reflections Interactive, Newcastle upon Tyne, UK) to examine the effectiveness of errorless learning in comparison to traditional trial-and-error on route learning. The VE was displayed in a 21-inch TV screen and interaction was facilitated with a control pad that was operated by an experimenter. Participants (eight TBI, 6 stroke, and 6 with brain tumors or cortical cysts) were required to learn a route using both techniques and repeat it afterwards. Significant differences were found between the number of errors made under both conditions, with errorless training showing less errors (Lloyd et al., 2006, 2009). In another study, Sorita et al. (2013) examined the route learning ability of 27 individuals with TBI. Participants were allocated in one of two groups and were required to learn a route in either a real (*n* = 13) or a virtual scenario (*n* = 14). The VE simulated a virtual street without cars or pedestrians, was projected into a 2.5 m wide and 1.8 m tall screen and was interacted with a gamepad. Both groups had to recall the route twice in the corresponding scenario immediately after the experiment and another time after 24 to 48 h. In addition, participants had to draw a sketch map of the route, select the correct map that represented the route among four possible options, and finally, arrange 12 pictures of the route in chronological order. Results showed no significant differences between both groups but in the arrangement task, where subjects who practiced in the real environment had better performance (*p* = 0.01) (Sorita et al., 2013).

In a different approach to street VE, Park (2015) compared the incidence of driving errors among 30 participants with left or right hemispheric lesions due to stroke. Driving errors were assessed using a VR driving simulator (GDS-300, Gridspace). The test course simulated driving in downtown Seoul and on the highway and was designed to resemble actual driving, incorporating various buildings, moving cars, traffic signals, and road signs. Significant differences were shown in center line crossing frequency, accident rate, brake reaction time, total driving error scores, and overall driving safety between participants with left or right hemispheric lesions, corroborating that rehabilitation specialists should consider hemispheric function when teaching driving skills to stroke survivors (Park, 2015). Spreij et al. (2020a), have also used a simulated driving task to assess differences in driving performance between patients with left- (*n* = 33) and right-sided VSN (*n* = 7), recovered VSN (*n* = 7), without VSN (*n* = 53), and HC (*n* = 21), as well as measuring VSN severity; and the driving simulator performance diagnostic accuracy in comparison to traditional tasks. Stroke patients were tested with a cancellation task, the Catherine Bergego Scale and the simulated driving task, which consisted of a straight road without intersections or oncoming traffic projected on a large screen. Participants were seated in front of the screen with a steering wheel fixed on a table. The simulated driving speed was approximately 50 km/h and participants were instructed to use the steering wheel to maintain the starting position at the center of the right lane, which demanded participants to adjust their position continuously. When participants drove off the road, the projection vibrated as a warning sign. Patients with left-sided VSN and recovered VSN deviated more regarding position on the road compared to patients without VSN. The deviation was larger in patients with more severe VSN. Regarding diagnostic accuracy, 29% of recovered VSN patients and 6% of patients without VSN did show abnormal performance on the simulated driving task. The sensitivity was 52% for left-sided VSN (Spreij et al., 2020a).

Finally, Ettenhofer et al. (2019) developed the Neurocognitive Driving Rehabilitation in Virtual Environments (NeuroDRIVE), an intervention designed to improve cognitive performance, driving safety, and neurobehavioral symptoms in TBI patients. The authors used the General Simulation Driver Guidance System (Driving assessment and training method and apparatus, 2014). This simulator consisted of an 8-foot circular frame supporting a curved screen (180° field of view) and a driving console analogous to that found in a typical automobile. The driving console had turn signals, gas and brake pedals, a steering wheel, digital dashboard, and a seat belt. Participants sat in the console and operated the steering wheel and pedals while responding to the VE projected onto the screen and auditory stimuli from connected speakers. The authors conducted a feasibility study to examine the preliminary efficacy of NeuroDRIVE. The intervention consisted of six 90-min sessions that included: (1) a brief review of training and progress thus far; (2) practice of component cognitive skills such as dual processing, working memory, and response inhibition through the use of standardized cognitive driving scenarios; (3) practice of composite driving skills such as following the rules of the road and being vigilant for road hazards while simultaneously performing working memory or visual attention tasks; and (4) finishing with an open-ended race-track course to promote engagement in the process and to allow participants to safely “test the limits” of their skills in a simulated environment. Eleven participants who received the intervention were compared to six waiting-list participants on driving abilities, cognitive performance, and neurobehavioral symptoms. The cognitive assessment protocol was the following: WAIS-III Digit Span, Symbol Search and Coding (Wechsler, 1997b), TMT A and B (Reitan, 1958), Controlled Oral Word Association Test (Letters & Animals), California Verbal Learning Test (CVLT) (Delis et al., 1988); Grooved Pegboard (Ruff and Parker, 1993), Neurobehavioral Symptom Inventory (Cicerone and Kalmar, 1995), PTSD Checklist-Civilian (Ruggiero et al., 2003), Beck Depression Inventory-II (BDI-II) (Beck et al., 1956), Epworth Sleepiness Scale (Johns, 1991), Fatigue Severity Scale (Krupp et al., 1989), Short Form Health Survey-36 (SF-36) (Ware, 1993), and Satisfaction with Life Scale (Diener et al., 1985). Participants that performed the NeuroDRIVE intervention had significant improvements in working memory and selective attention, two of the primary objectives of the intervention. There was no generalization of improvements to other cognitive domains, neurobehavioral symptoms or driving skills (Ettenhofer et al., 2019).

### 3.5. Cities

Virtual cities have been considered in the literature to recreate daily living activities that involve moving around a wide area and visiting different locations to perform cognitive-demanding tasks that require basic cognitive functioning (Gamito et al., 2011a,b, 2014, 2015; Jovanovski et al., 2012; Vourvopoulos et al., 2014; Faria et al., 2016, 2019, 2020; Oliveira et al., 2020; Table 5). A total of eleven studies simulating cities have been included in this review, which involved 209 participants with stroke, five participants with TBI, one Mild Cognitive Impairment (MCI) and 74 healthy participants. Designs included four RCT’s, two feasibility studies, two validation studies, a case study, a pilot study, and a usability study.

Gamito and colleagues (2011) developed a small virtual town populated with several characters and buildings arranged in eight squared blocks, along with a two-room apartment and a mini-market. The VE was displayed in an HMD and interaction, based on moving around and grabbing objects, was enabled using a keyboard and a mouse. The VE required participants to perform daily activities such as finding a supermarket and buying some items, or finding and retaining paths, characters, or advertisements. The potential of the VR system to improve attention and memory after TBI was investigated in a case study consisting on nine sessions administered with unknown frequency, with a resulting mean duration of 45 min each. The participant was assessed with the Paced Auditory Serial Addition Task (Gronwall, 1977) before, during (after the fifth session), and after the intervention, with three and two-second inter-stimulus intervals. The results showed a significant increase in the percentage of correct responses between the previous and intermediate assessment for both trials and between the intermediate and final assessment (Gamito et al., 2011a). A similar procedure with the same VE and instrumentation, consisting on nine weekly sessions of unspecified duration, was replicated with two individuals with stroke, whose memory and sustained attention were assessed with the WMS-III (Wechsler, 1997a) and the Toulouse Piéron (TP) (Piéron, 1955), respectively (Gamito et al., 2011b). Like the previous study, the results revealed increased memory and attention capabilities after the intervention, although no statistical analysis could be performed. A later study by the same authors with the same system compared the impact of two possible displays, an HMD and a 21-inch monitor, on the effectiveness of the intervention (Gamito et al., 2014). Seventeen individuals with stroke were randomized into an HMD (*n* = 8) or a monitor (*n* = 9) display condition, and underwent the same intervention. In this later study, participants were also assessed with the Rey-Osterreith Complex Figure (ROCF) (Osterrieth, 1954). Results evidenced a significant improvement in the WMS-III (*p* < 0.01), the ROCF (*p* < 0.05), and the TP (p < 0.01) in both groups. However, no statistical differences emerged between the two conditions, which suggests that both HMD and screens could be valid alternatives for providing visual feedback during VR-based interventions on memory and attention. A RCT involving 20 individuals with stroke compared the effectiveness of a 4 to 6-week intervention with the same system including two to three 60-min sessions per week (*n* = 10) in comparison to HC (*n* = 10), consisting of a waiting list (Gamito et al., 2015). Participants were assessed with the same tests and procedure of the previous study. In contrast to the control group, who showed no improvement, the experimental group significantly improved their scores in the WMS-III and the TP, giving rise to significant differences between groups in both tests. No differences in time or between groups were detected in the ROCF. Subsequently, Oliveira et al. (2020) also performed a study to test this ecologically oriented approach, depicting everyday life tasks (Systemic Lisbon Battery), in a sample of 30 sub-acute stroke inpatients in a rehabilitation hospital. Participants were assessed in a single-arm pre-post intervention study revealing improvements on global cognition with the Montreal Cognitive Assessment (MoCA), executive functions with the FAB, memory with the WMS-III memory quotient and attention with the CTT execution time reduction (Oliveira et al., 2020).

With a more global approach, Jovanovski et al. (2012) developed the Multitasking in the City Test and investigated its convergent validity with a battery of neuropsychological tests in a sample of eleven stroke and two TB participants (Jovanovski et al., 2012). The VR system consists of eleven different buildings (from a post office to an optometrist’s office) and the participant’s home. Virtual elements were displayed in a computer monitor to be interacted with a joystick. Participants were required to purchase several items, obtain money from the bank, and attend a doctor’s appointment within a period of 15 min. Performance in the VR-based system was evaluated according to the completion time, tasks completed, task repetitions, insufficient funds, inefficiencies and task failures. Clinical testing included the Controlled Oral Word Association Test (COWAT) (Benton and Hamsher, 1989), Semantic Fluency (Animals), WCST (Berg, 1948), BADS (Wilson et al., 1996), TMT (Reitan, 1958), WAIS-III (Wechsler, 1997b), Judgment of Line Orientation (Benton et al., 1975), ROCF (Osterrieth, 1954), CVLT (Delis et al., 1988), and Wechsler Memory Scale-III (Wechsler, 1997a). With regards to executive functioning, good to excellent correlations emerged between the TMT Part B and the VE tasks completion time (*r* = 0.64, *p* = 0.02) and between the WCST and VE tasks completion time (*r* = 0.84, *p* < 0.01) and total errors (*r* = 0.60, *p* = 0.03). Moderate correlations were found between the TMT Part A and the VE tasks completion time (*r* = 0.59, *p* = 0.04) and between the Judgment of Line Orientation test and the VE tasks total errors (*r* = −0.56, *p* = 0.05) and completion time (*r* = −0.59, *p* = 0.03).

Vourvopoulos et al. (2014) developed the Reh@City, a virtual city that simulates several ADLs within a supermarket, a post-office, a pharmacy, and a bank, which aimed to train visuospatial orientation, attention, and executive function (Vourvopoulos et al., 2014). The VE was displayed in a 24-inch computer monitor, and a joystick enabled navigation and interaction. Participants were engaged in multiple tasks that involve visuospatial orientation (to navigate to appropriate places), attention (to select target elements among distractors), and executive function (to buy groceries or to withdraw cash euros from an ATM machine). Performance in the VR-based system was assessed according to the score, distance, and time spent in navigation or in task completion. In a preliminary study, the authors investigated the convergent validity of the Reh@City with the MMSE (Folstein et al., 1975) and the Stroke Impact Scale (SIS) (Duncan et al., 2003). A one-session pilot study with ten post-stroke participants (7 stroke, 2 TBI and 1 MCI) revealed a strong correlation between the score and distance traveled in the Reh@City with the MMSE (*r* = 0.81, *p* < 0.05, and *r* = 0.65, *p* < 0.05, respectively). In addition, mood stability and the control item of the SIS also showed good correlations with scores (*r* = 0.75, *p* < 0.05) and time spent in navigation (*r* = −0.72, *p* < 0.05) and task completion (*r* = 0.72, *p* < 0.05). A later RCT study with the Reh@City, carried by the same authors, included 18 post-stroke participants who were randomly assigned to an experimental training with the system (*n* = 9) or to a conventional intervention consisting on occupational therapy sessions (*n* = 9) (Faria et al., 2016). Participants underwent twelve 20-min sessions distributed from 4 to 6 weeks and were assessed with the Addenbrooke Cognitive Examination (ACE) (Mioshi et al., 2006), the TMT Part A and B, the Picture Arrangement subtest of the WAIS-III, and the SIS. Although both groups improved in almost all tests and subtests of the neuropsychological assessment battery, greater improvements were detected in those participants who trained with the VR system in the total score, and attention and fluency subtests of the ACE, and also in the MMSE. Subsequently, authors implemented a personalization and adaptation framework (Faria et al., 2018) in a Reh@City 2.0 version, also increasing the variety of locations for ADL’s simulations: magazine kiosk, fashion store, park, home. Reh@City 2.0 was compared with a content equivalent paper-and-pencil cognitive training tool, which follows the same personalization and adaptation framework, in an RCT with 36 stroke patients (Faria et al., 2020). The intervention comprised 12 sessions, with a neuropsychological assessment pre, post-intervention and follow-up, having as primary outcomes: general cognitive functioning (MoCA), attention (TMT Part A and B), memory (Verbal Paired Associates from WMS-III), executive functions (Digit-Symbol Coding, Symbol Search and Digit Span from WAIS-III) and language (Vocabulary from WAIS-III) specific domains; and as secondary outcome the self perceived impact of cognitive deficits in different aspects of everyday life (everyday life skills, family and life, mood and sense of self), measured by the Patient Reported Evaluation of the Cognitive State (PRECiS) (Patchick et al., 2015). Results revealed that the Reh@City v2.0 improved general cognitive functioning, attention, visuospatial ability and executive functions. These improvements generalized to verbal memory, processing speed and self-perceived cognitive deficits specific assessments. The paper-and-pencil intervention only had impact in the MoCA orientation domain, processing speed and verbal memory outcomes. However, at follow-up, processing speed and verbal memory improvements were maintained, and a new one was revealed in language. Between-groups, the Reh@City v2.0 was superior in general cognitive functioning (Faria et al., 2020). The authors also analyzed the session-to-session performance in this intervention in order to compare the paper-and-pencil with the ecologically valid VR-based approach (Faria et al., 2019). Results have shown that both groups performed at the same level and there was not an effect of the training methodology in overall performance. However, the Reh@City enabled a more intensive training, which may translate in more cognitive improvements.

Claessen et al. (2016) compared spatial navigation in real world and in a virtual city, which consisted on a photorealistic virtual rendition of a real city. Both routes were about 400 m and involved 11 decision points, where participants decided to take a left or right turn. A sample of 68 stroke participants and 44 HC navigated within a real and the virtual city, after which they completed eight subtasks addressing route knowledge (scene recognition, route continuation, route sequence and order) and integration of geometrical aspects (distance and duration estimation, route drawing, and map recognition). Significant poor to moderate correlations between virtual and real navigation were found for HC and stroke participants in route continuation (*r* = 0.27, *p* = 0.27, and *r* = 0.37, *p* = 0.013, respectively), order (*r* = 0.35, *p* = 0.03, and *r* = 0.31, *p* = 0.043, respectively), and distance estimation (*r* = 0.31, *p* = 0.12, and *r* = 0.56, *p* < 0.001, respectively). An additional significant poor correlation was found in the patients group for route sequence (*r* = 0.27, *p* = 0.029) (Claessen et al., 2016).

### 3.6. Other everyday life scenarios

Other everyday life scenarios have been simulated in VR for cognitive rehabilitation (Table 6). In this section, 10 studies have been included (3 RTC’s, 5 pilot study, 1 usability study, and 1 prospective cross-sectional study) involving 114 stroke, 116 TBI, two brain tumour, 11 MS, 87 HC, 12 intracerebral hemorrage, 1 encephalitis and 6 degenerative disease participants.

Targeting the assessment of executive functions, Renison et al. (2012) developed the Virtual Library Task, a virtual replica of a real library where participants are required to perform different tasks associated to daily routine in a library (Renison et al., 2012). Participants must prioritize and complete tasks, such as cooling down the library or checking items that appear in the in-tray, while managing interruptions and acquisition of new information. The VE is displayed in a computer monitor and is interacted using a gamepad. The authors compared the performance of 30 TBI and 30 HC participants in the virtual and the real life scenario in two different 90-min sessions and were also assessed with the Verbal Fluency Test (Alderman et al., 2003), the WCST (Heaton, 2005), the Brixton Spatial Anticipation Test (Burgess and Shallice, 1996), the Zoo Map and MSET from the BADS (Wilson et al., 1996). Results showed that performance on the virtual and real tests had significant weak to moderate correlations that were evidenced in the total score (*r* = 0.68, *p* > 0.01) and scores of the subtests, which included task analysis (*r* = 0.27; *p* = 0.04), strategy generation and regulation (*r* = 0.77; *p* = 0.01), prospective working memory (*r* = 0.53; *p* = 0.01), response inhibition (*r* = 0.54; *p* = 0.01), and timed (*r* = 0.48; *p* = 0.01) and event-based prospective memory (*r* = 0.73; *p* = 0.01). Although both groups had similar cognitive condition, with only one difference in the MSET (*p* = 0.02), TBI participants performed significantly worse than HC in the VE, which drew significant differences for total score, prospective working memory, dual tasking, and timed and event-based prospective memory (Renison et al., 2012).

With the same objective of the pervious group, Krch et al. (2013) developed the Assessim Office, a virtual office that aims to evaluate executive functioning from the performance on tasks that simulate real word demands, such as responding to emails while ensuring that a projector remains on (selective and divided attention), decision-making on real estate offers (problem solving), printing offers (working memory), and delivering printed offers to a file box (prospective memory) (Krch et al., 2013). The VE was shown on a computer monitor with stereo desktop speakers, and navigated and interacted using a two-key mouse. In a pilot study, the authors compared the performance of seven TBI individuals with that of seven HC (and five individuals with multiple sclerosis) and explored the relationship between the performance of the neurological participants with neuropsychological measures of executive function, which included the Letter Number Sequencing and Digit Span Backwards of the WAIS-III (Wechsler, 1997b), the Color Word Test of the Delis-Kaplan Executive Function System (Delis et al., 2000), and the Wechsler Abbreviated Scale of Intelligence (WASI) (Axelrod, 2002). Overall, HC had better performance than participants with TBI, but significant differences were only detected for the correct real estate decisions. Exceptionally, participants had better performance at printing declined offers. Excellent correlations were detected between the raw score and set loss errors of the Delis-Kaplan Executive Function System and the incorrect prints (*r* = −0.889, *p* = 0.044; and *r* = −0.913, *p* = 0.030, respectively) and projector light misses (*r* = 0.947, *p* = 0.014; and *r* = 0.973, *p* = 0.005, respectively). The color word inhibition and inhibition/switching scores of the same test had excellent correlations with the number of emails correctly replied (*r* = −0.900, *p* = 0.037) and offers delivered (*r* = 0.894, *p* = 0.041) (Krch et al., 2013). Matheis et al. (2007) had also previously developed a VR-based office to assess learning and memory in TBI. The authors compared 20 TBI participants with 20 HC on their ability to learn and recall 16 target items presented within a VR-based office environment through a 5th Dimension Technologies 800 Series HMD. Besides the VR-based learning and memory task initial acquisition within 12 learning trials, short-term recall (30 min.) and long-term recall (24 h) performance, outcome measures consisted in a complete battery of neuropsychological measures, and the modified Simulator Sickness Questionnaire (m-SSQ) (Kennedy et al., 1993). The following neuropsychological measures were applied: the WASI (Axelrod, 2002); the digit span and digit symbol-coding subtests from the WAIS-III (Wechsler, 1997b); the TMT Part A and B (Reitan, 1958); the WCST (Heaton, 1981); the Boston Naming Test (BNT) (Kaplan et al., n.d.); the Hooper Visual Organization Test (Hooper, 1983); the COWAT (Spreen and Strauss, 1998); the CVLT (Delis et al., 1988); and the Brief Visuospatial Memory Test-Revised (Benedict et al., 1996). The results indicated that VR memory testing accurately distinguished the TBI group from controls. Additionally, non-memory-impaired TBI participants acquired targets at the same rate as HC participants. Finally, the authors found a significant relationship between the VR Office and a standard neuropsychological measure of memory, suggesting the construct validity of the task (Matheis et al., 2007).

Also for executive functions assessment, Gilboa et al. (2017) developed the Jansari assessment of Executive Functions for Children (JEF-C) and tested its feasibility and validity in children and adolescents with ABI. In the JEF-C the participant has to plan, set up and run their birthday party through the completion of tasks. The party takes place in a virtual home with three rooms, the kitchen, the living room and the DVD/games room. The participant can move freely around with the computer mouse. Realistic tasks that could happen in a birthday party have been created in order to ecologically tackle eight constructs: planning; prioritization; selective, adaptive and creative thinking and; action, time and event-based prospective memory. All participants (29 ABI patients from 10 to 18 years +30 age-and gender-matched controls) performed the JEF-C, the WASI and the BADS for Children (BADS-C), while parents completed the Behavior Rating Inventory of Executive Function questionnaire. The JEF-C task proved feasible for ABI children and adolescents. The internal consistency was medium (Cronbach’s alpha = 0.62 and significant intercorrelations between individual JEF-C constructs). Patients performed significantly worse than controls on most of the JEF-C subscales and total score, with 41.4% of participants with ABI classified as having severe executive dysfunction. No significant correlations were found between JEF-C total score, the BRIEF indices, and the BADS-C. Significant correlations were found between JEF-C and demographic characteristics of the sample and intellectual ability, but not with severity/medical variables. JEF-C appears to be a sensitive and ecologically valid assessment tool, especially for relatively high-functioning individuals (Gilboa et al., 2019).

With a wider goal of cognitive assessment and rehabilitation, Fong et al. (2010) developed a virtual replica of an ATM to train three common tasks: cash withdrawal, money transfer, and electronic payment (Fong et al., 2010). In a first experiment, the authors compared the sensitivity and specificity of the cash withdrawals and money transfers tasks. A sample of nine stroke and five TBI participants performed the tasks in the real and the virtual ATMs. The sensitivity of the virtual replica was 100% for cash withdrawals and 83.3% for money transfers, and the specificity was 83 and 75%, respectively. In a second experiment, nine participants with stroke and a participant with a brain tumor were assigned in matched pairs to either a VR-based training with the virtual ATM or a computer-assisted instruction teaching program with multimedia tutorials with feedback and verbal reinforcement for six 1-h sessions over a three-week period. Participants’ general cognitive condition was also assessed with the Neurobehavioral Cognitive Status Examination (Chan et al., 2002). Results showed better performance of participants who trained with the virtual ATM in reaction time (*p* = 0.021) and score (*p* = 0.043) in the virtual cash withdrawal task after treatment, but not in the money transfer task (Fong et al., 2010).

Gerber et al. (2014) simulated some interactive virtual tasks that were displayed in a computer monitor and interacted through the haptic device Phantom® Omni™ (3D Systems, CA, USA) (Gerber et al., 2014). Tasks included removing tools from a workbench, composing 3-letter words, preparing a sandwich, and hammering nails. Nineteen individuals with TBI were enrolled in usability study and interacted with each task three times for a maximum of 5 min (2 min in the word composing task) and were assessed with the Boredom Propensity Scale (Farmer and Sundberg, 1986), the Purdue Pegboard Test (Mathiowetz et al., 1985), the Neurobehavioral Symptom Inventory (Cicerone and Kalmar, 1995), and the Wolf Motor Function Test (Wolf et al., 2001). Moderate correlations were detected between clinical scales and scores of the third iteration of the tasks. Specifically, the time to complete the workbench clearance and the hammering task correlated with the Purdue Pegboard Test (*r* = −0.652, *p* = 0.016; and *r* = −0.598, *p* = 0.014, respectively), and the number of words completed correlated with the Neurobehavioral Symptom Inventory (*r* = −0.494, *p* = 0.052). In addition, according to the Boredom Proneness Scale, all participants were highly engaged in the interaction (Gerber et al., 2014).

Focusing on upper-limbs training, Fluet et al. (2013) involved 30 individuals with stroke in a study to compare the effectiveness of a virtually simulated program of repetitive task practice with a comparable program of conventionally presented activities (Fluet et al., 2013). The VE simulated real life activities such as reaching items, placing cups, hammering, and playing piano, with other fictional narratives. Visual feedback was provided using a monitor and interaction was facilitated through a CyberGlove (Immersion, USA), an instrumented glove for measuring finger angles, which was equipped with a CyberGrasp (Immersion, USA) that provided haptic feedback, and a Haptic MASTER (Moog NCS, The Netherlands), a force controlled robot with three degrees of freedom. Conventionally presented activities included reaching items, writing, keyboarding, cooking, dressing, etc. Participants performed one of the two programs for eight 3-h sessions in a two-week period and were assessed before and after the intervention, and 3–6 months after with the Upper Extremity subscale of the FMA (Fugl-Meyer et al., 1975), the Wolf Motor Function Test, and the Jebsen Test of Hand Function. Both groups evidenced improvements with time in the three scales that reached statistical significance, which was not specified in the text. However, no statistically significant differences between groups were detected at any of the three measurement times.

Also for upper-limbs rehabilitation, Qiu et al. (2020) developed the Home based Virtual Rehabilitation System (HoVRS) and performed a feasibility study with 15 chronic stroke participants. HoVRS was placed in participants’ homes that were asked to use the system at least 15 min every weekday for 3 months (12 weeks) with limited technical support and remote clinical monitoring. The intervention included a subset of 5 games (Maze, Wrist Flying, Finger Flying, Car, Fruit Catch) out of the HoVRS 12-game library, at least one from type of movement category (Elbow-Shoulder, Wrist, Hand, Whole Arm). Each weekday, subjects were encouraged to play at least 3 rehabilitation activities for a minimum of 15 min. Participants were assessed pre and post intervention with the Upper-Extremity FMA. In addition, six-hand and arm kinematics were measured using testing games and subsequently analyzed. Participants were able to complete the study without any adverse events and spent on average 13.5 h using the system. At post intervention participants demonstrated a mean increase of 5.2 on the FMA and improved in six measurements of hand kinematics. Additionally, a combination of these kinematic measures was able to predict a substantial portion of the variability in the subjects’ UEFMA score (Qiu et al., 2020).

Cho and Lee (2019) investigated the impact of VR immersive training with computerized cognitive training with the RehaCom (Schuhfried, 1996) on the cognitive function and ADLs in acute stroke patients. The patients were randomly divided into the experimental (*n* = 21) and control (*n* = 21) group. The experimental group performed VR training with a HMD with computerized cognitive therapy (RehaCom), and the control group performed computerized cognitive therapy (RehaCom). The VR training consisted in Fishing and Picture Matching tasks, in the first the user had to catch fish using upper extremities, and in the second, the user had to flip cards and find a match, the initial screen had 8 cards and the user could turn or look back to see all the cards. All participants trained for 30 min a day 5 times a week and the intervention lasted 4 weeks. To evaluate the improvement in each group, pre-post-test evaluation was conducted using the Loewenstein Occupational Therapy Cognitive Assessment (LOTCA) (Katz et al., 1989), the Computerized Neurocognitive Function Test (CNT) (Kwon et al., 2002), and the FIM (Grimby et al., 1996). For changes before and after the intervention in the CNT, the experimental group was significantly superior in the Auditory Continuous Performance Test (ACPT) from the CNT (experimental = 10.24, control = 3.29, *p* = 0.01), in the VRT (experimental = 1.76, control = 0.76, *p* < 0.00) and in VRT-recall (experimental = 1.81, control = 0.81, *p* < 0.00). For FIM total motor function, the experimental group was also superior (experimental = 19.19, control = 9.43, *p* < 0.00). The experimental group showed significant improvements in all LOTCA, CNT and FIM items from pre to post intervention (Cho and Lee, 2019).

Finally, in a leisure approach, Lorentz et al. (2021) conceptualized the VR Traveller, a training program of attentional functions in a more engaging setting that also resembles real-life activities. The program offers several modules to address specific attentional dysfunctions within the context of a virtual journey around the world. Each scenario is set in a different location and addresses alertness, selective attention, visual scanning and working memory. The VR Traveller training program was completed by 35 patients with ABI in a 20–30 min session during inpatient neurorehabilitation. Feasibility and acceptability were assessed with the user experience questionnaire (UEQ) (Laugwitz et al., 2008) and a self-constructed feasibility questionnaire, and tolerability was assessed with the virtual reality sickness questionnaire (VRSQ) (Kim et al., 2018). Overall, patients’ ratings of the VR training in terms of acceptability and feasibility were positive, suggesting that VR programs represent an accepted, feasible, and well-received alternative to traditional cognitive rehabilitation approaches (Lorentz et al., 2021).

## 4. Discussion

VR technologies have evolved from rudimentary systems in the 1960s to sophisticated immersive environments in the last decades. VR has been increasingly used as a tool for neuropsychological assessment and rehabilitation. The purposefulness of this review was to provide an overview about the use of ecologically valid virtual environments and related technologies to assess and rehabilitate people with ABI. In this section we discuss our findings according to the objectives stated in the Introduction.

### 4.1. What are the most common virtual environments used in acquired brain injury assessment and rehabilitation?

With this revision we have identified the main daily life environments and tasks that are simulated through VR. Overall, we have considered 70 studies, in which 12 were simulations of kitchens, 11 supermarkets, 10 shopping malls, 16 streets, 11 cities and 10 other everyday life scenarios.

In light of the existing studies, we have concluded that virtual kitchens may have the potential to discriminate between healthy and pathological performance and to simulate meal and hot drink preparation tasks with moderate correlations to real life performance (Zhang et al., 2003; Edmans et al., 2006; Besnard et al., 2016; Triandafilou et al., 2018; Thielbar et al., 2020) and neuropsychological (Besnard et al., 2016) and motor tests (Adams et al., 2015; Huang et al., 2017).

Considering the supermarket context, different studies have shown moderate correlations between the performance of HC and post-stroke participants in virtual supermarkets with neuropsychological tests (Josman et al., 2006, 2014; Raspelli et al., 2012; Yip and Man, 2013; Sorita et al., 2014; Cogné et al., 2018). Moderate correlations were also evidenced between the performance in real and virtual shopping tasks (Yip and Man, 2013). Performance in virtual supermarkets has been also shown to successfully differentiate between healthy and post-stroke participants (Kang et al., 2008; Raspelli et al., 2012; Cogné et al., 2018; Ogourtsova et al., 2018). Moreover, virtual supermarkets have also been effectively used to train upper limb motor function with similar effectiveness to conventional therapy, although effectiveness of the intervention is expected to rely on the motor task rather than the environment (Yin et al., 2014; Demers and Levin, 2020). Finally, a VR kitchen was presented in a computer monitor and a HMD and both stroke patients and HC reported an enhanced feeling of engagement, transportation, flow, and presence in the HMD condition (Spreij et al., 2020b).

Also within a shopping task perspective, virtual malls are widely used to assess and rehabilitate ABI patients. Existing literature shows a general sensitivity of the virtual tasks recreated in virtual malls to differentiate between healthy individuals and individuals with stroke (Rand et al., 2007, 2009a; Hadad et al., 2012; Okahashi et al., 2013; Nir-Hadad et al., 2015) and TBI (Canty et al., 2014), and between young and older adults (Rand et al., 2009a; Okahashi et al., 2013). Virtual tasks also showed moderate to high correlations with clinical tests in stroke (Nir-Hadad et al., 2015) and TBI (Erez et al., 2013; Okahashi et al., 2013; Canty et al., 2014), and more importantly, moderate correlations with performance in mockups (Hadad et al., 2012; Nir-Hadad et al., 2015) and in real world scenarios (Rand et al., 2009b; Hadad et al., 2012; Nir-Hadad et al., 2015). Interventions with VEs recreating virtual malls showed preliminary effectiveness at improving upper limb function (Rand et al., 2009a), as well as multitasking (Rand et al., 2009c), and other executive functions (Jacoby et al., 2013).

Street crossing and driving are demanding tasks that are commonly impaired after brain injury. Studies suggest worse performance of individuals with ABI as both virtual drivers (Liu et al., 1999) and pedestrians aiming to cross the street (Naveh et al., 2000; Weiss et al., 2003; Navarro et al., 2013), with increased difficulties in presence of USN (Navarro et al., 2013), and to remember a route (Titov and Knight, 2005) in comparison to healthy subjects. Street crossing has shown concurrent validity with standardized neuropsychological tests (Navarro et al., 2013), which was not replicated with virtual driving (Wald et al., 2000). Moreover, virtual street crossing, driving, and walking can be used to improve neglect, the ability to drive, and route retaining, with comparable efficacy to visual scanning tasks (Katz et al., 2005), driving-related tasks (Akinwuntan et al., 2005), or walking in the real world (Sorita et al., 2013), respectively. However, training in more ecological conditions could provide increased visual and anticipation abilities (Devos et al., 2009), which could have a positive transference to driving in the real world (Akinwuntan et al., 2005), and to episodic memory (Sorita et al., 2013), working memory and selective attention (Ettenhofer et al., 2019). In terms of assessment, VR street crossing systems are potentially useful to differentiate USN and non-USN individuals (Spreij et al., 2020a; Wagner et al., 2021).

Virtual cities allow for a diversity of everyday life tasks simulations. The here presented studies showed convergent validity between some measures of the performance in simulated virtual cities and clinical neuropsychological tests of variable strength, which ranges from moderate for attention (Jovanovski et al., 2012), good to excellent for executive functioning (Jovanovski et al., 2012), and excellent for general cognitive condition (Vourvopoulos et al., 2014; Oliveira et al., 2020) and even mood (Vourvopoulos et al., 2014). Poor to moderate correlations has been also reported between navigation in a real and a virtual city (Claessen et al., 2016). Training in virtual cities has been also shown to improve processing speed, flexibility, and calculation after TBI (Gamito et al., 2011a), and attention and memory after stroke (Gamito et al., 2011b, 2014). Remarkably, effectiveness of VR-based training in virtual cities after stroke has been also reported not only in comparison to no intervention, the former providing greater improvements in attention and memory (Gamito et al., 2015), but also in comparison to matched conventional occupational therapy, the former providing greater improvements in general cognitive condition, attention and fluency (Faria et al., 2016). Even when compared to a time-matched content equivalent paper-and-pencil training, a more ecological valid training with a virtual city revealed higher effectiveness with improvements in different cognitive domains and self-perceived cognitive deficits in everyday life (Faria et al., 2020). It should be also highlighted that similar improvements have been provided with immersive and non-immersive displays, showing little effect of the enabling technology (Faria et al., 2019, 2020).

Ultimately, simulation of tasks in other everyday life environments, such as virtual libraries, offices, ATMs, workbenches, virtual travelling etc., have been reported to have certain sensitivity to impairments after TBI in memory (Renison et al., 2012) and executive functions (Renison et al., 2012; Krch et al., 2013), and also good sensitivity and specificity to predict performance in simples tasks in the real world (Fong et al., 2010). Convergent validity between the performance in the virtual tasks has been reported to be excellent with clinical measures of executive function (Krch et al., 2013; Gilboa et al., 2019), general cognitive functioning (Cho and Lee, 2019), moderate with neurobehavioral symptoms (Gerber et al., 2014), and good with measures of hand dexterity (Gerber et al., 2014). In addition, performance in the virtual world has also shown moderate to good convergent validity with some measures of memory and executive function during performance in the real world (Renison et al., 2012). Finally, training with VE simulating other environments has been shown to be specific, thus improving the performance in the virtual task in comparison to a computer-assisted instruction teaching program (Fong et al., 2010) and provide comparable improvements on upper-limb motor function assessed in comparison to conventionally presented activities (Fluet et al., 2013; Qiu et al., 2020). Finally, it is important to highlight that leisure simulations, such as travelling, are also promising for assessment and rehabilitation of ABI (Lorentz et al., 2021).

### 4.2. Which technologies are used for presentation and interaction In these environments?

The VEs that were presented in this review were mostly presented in computer screens (26 studies), HMD’s (16 studies), laptops (six studies) and wall projections (two studies), and patients interacted with them primarily via mouse (19 studies), keyboard (15 studies), joystick (nine studies), GestureTek (6 studies) Kinect (six studies) and touchscreen (five studies).

### 4.3. How are these virtual environments being clinically validated about their impact in ABI assessment and rehabilitation?

According to this review, a great number of ecologically valid simulations of daily-life tasks mostly target cognitive domains, such as general executive functions (25 studies), attention (18 studies), memory (10 studies) and general cognition (eight studies). Only 11 studies focused in motor aspects assessment and rehabilitation.

Numerous studies have been carried out to clinically validate these VR-based assessment and rehabilitation environments, from case studies to RCTs. Evidence stills modest, and further research with more extensive and homogeneous samples is needed. In 70 studies 45 had an experimental design and 25 a non-experimental design.

There is also a huge need of major uniformity of the neuropsychological and motor tests that are used in these studies to strengthen conclusions and allow comparison between studies. In the universe of 70 studies, 136 different outcome measures are used for the following main domains: cognitive, functional, motor, emotion, cybersickness, immersion and engagement. The 10 most used instruments and questionnaires were: the WAIS-III for general cognition (13 studies), the MMSE cognitive screening (13 studies), the BADS for executive functions (10 studies), the TMT Part A and B for attention, processing speed and working Memory (eight studies), WMS-III for general memory (eight studies), WCST also for executive functions (seven studies), the WAIS Digit Span for Memory and Working Memory (seven studies), the FIM for general functionality (six studies), the FMA for motor functions (seven studies) and the BDI-II for depressive symptomatology (five studies).

Although higher levels of engagement is allegedly one of the VR tools advantages, only one study has assessed it (O’Brien, 2007). Additionally, only a marginal number of studies (nine) assessed presence and immersion, the most used outcome measure in this domain (four studies) was the SFQ (Witmer and Singer, 1998).

### 4.4. Implications for clinical practice

One of the unresolved issues that must be addressed is the suitability of particular VR platforms in relation to the therapeutic goals one wishes to achieve. In total, 12 different self-report functional assessment scales were used as outcome measures, demonstrating a likely direct transfer from the performance in ecologically valid VR tasks to everyday life tasks routines. Health professionals should consider this transfer effect when choosing a test for assessing the capacity of driving or training tasks that address rehabilitation (Krasny-Pacini et al., 2016).

One of the critical issues in VR-based neuropsychological assessment is the targeted end-user (i.e., the person who uses it as an assessment tool). As defined by the American Psychological Association (APA), researchers and clinicians “do not promote the use of psychological assessment techniques by unqualified persons, except when such use is conducted for training purposes with appropriate supervision” (Campbell et al., 2010), Ethical Standard 9.07, Assessment by Unqualified Persons). VR instruments can be implemented by other professionals who do not have a background in neuropsychology but the results should be integrated and interpreted by a competent professional such as a neuropsychologist. As such VR-based assessment instruments should be defined to be administered by a clinician or researcher who has competency in neuropsychological assessment and warranties privacy and data security, test scoring and interpretation, and record keeping (Kourtesis et al., 2020). Even though most VR-based tools do not require the intervention of a health professional, their supervision and guidance are essential not only in the assessment but also the recovery process (Bruno et al., 2022).

The time and frequency of interaction with immersive and non-immersive VR technologies is highly variable across the studies, clinical guidelines would be valuable. For instance, Kourtesis et al. (2019) findings support the viability of VR sessions with duration up to 70 min, when the participants are familiarized with VR technology and the quality of the VR software meets the parsimonious cut-offs of the Virtual Reality Neuroscience Questionnaire (VRNQ). This questionnaire was developed and validated by Kourtesis et al. (2019) to assess the quality of VR software in terms of user experience, game mechanics, in-game assistance, and cybersickness.

Finally, although there is no strong evidence that the use of VR is more beneficial than conventional therapy in ABI assessment and rehabilitation (e.g., Laver et al., 2017), this technological approach as demonstrated to be beneficial as complementary to usual care in the reviewed studies, for numerous reasons, namely: is more engaging (O’Brien, 2007); enables a more intensive training (Faria et al., 2019); provides immediate feedback (Fong et al., 2010); tasks have greater verisimilitude and validity (Nir-Hadad et al., 2015).

### 4.5. Implications for research

The lack of robust evidence on the clinical validity and impact of VR-based ecologically valid assessment and rehabilitation tools is mainly due to the need for studies of higher methodological quality (RCTs) and greater sample size. Moreover, in the case of rehabilitation, having an active control group is essential to confirm its clinical efficacy. In this review, only Faria et al. (2020) compared a VR-based intervention with its equivalent conventional approach (paper-and-pencil), although with a small sample size.

In terms of future research directions, it is important to mention the work from Kourtesis and colleagues who developed the Virtual Reality Everyday Assessment Lab (VR-EAL) (Kourtesis et al., 2019), the first immersive and ecologically valid VR neuropsychological battery, developed to meet the criteria of the National Academy of Neuropsychology (NAN) and American Academy of Clinical Neuropsychology (AACN) for Computerized Neuropsychological Assessment Devices (CNADs). The AACN and NAN recognize the potential advantages of CNADs as testing large numbers of individuals quickly (e.g., parallel administration); immediately available tests; enhanced accuracy and precision (e.g., reaction time measurements); shorter administration time and reduced costs (e.g., for test administration and scoring); adaptable in different languages; exporting the data automatically (e.g., for research purposes); increased accessibility (e.g., remotely); and the integration of algorithms for making decisions on issues such as the identification of an impairment or a statistically reliable change (Bauer et al., 2012). Since it was developed, the VR-EAL has already shown that it achieves several of these benefits. The VR-EAL is immediately available after its installation on a personal computer and automatically produces accurate performance scores, it has no costs for administration and scoring, and it requires a shorter administration time as compared to its equivalent paper-and-pencil counterparts. The VR-EAL is only missing a predictive algorithm for identifying cognitive impairment, since it has not been validated with any clinical population yet (Kourtesis and MacPherson, 2021). The work from these authors is an example of good practices and is, as such, an important reference for future studies in this field.

Another relevant topic for future research is the impact of the levels of immersiveness. The VEs that were explored in this review were mainly presented, in an ascending degree of immersiveness: laptops (six studies), computer screens (26 studies), wall projections (two studies) and HMDs (16 studies). Understanding how the level of immersion influences the effectiveness of the therapy or assessment will provide valuable insights and more nuanced recommendations for practice (Vasser and Aru, 2020). In this review only three studies measured presence and immersion, namely with the Presence Questionnaire (Witmer and Singer, 1998), the Igroup Presence Questionnaire (igroup.org – project consortium; igroup presence questionnaire (IPQ) overview | igroup.org – project consortium; IPQ, n.d.) and the Immersive Tendencies Questionnaire (Witmer and Singer, 1998).

Cybersickness is a significant side effect associated with the use of VR and arises from a conflict between visual, vestibular, and proprioceptive systems (Kourtesis et al., 2019). According to the literature, researchers and clinicians do not quantitatively assess cybersickness despite its impact on cognitive performance (Nesbitt et al., 2017; Arafat et al., 2018; Mittelstaedt et al., 2019). Indeed, in the universe of 70 studies only three have assessed cybersickness with objective measures, namely the Virtual Reality Sickness Questionnaire (Kim et al., 2018) and the Simulator Sickness Questionnaire (Kennedy et al., 1993). Understanding how to avoid or mitigate these symptoms is crucial for the safe and effective use of VR in neuropsychological assessment and rehabilitation.

Finally, evidence would be enriched if future studies in this field incorporate neuroimaging to explore neuroplasticity changes that positively affect the participants’ cognition, humor and functionality. Given the specificity of some VR-based scenarios (like kitchens and streets) and the multi-domains approach of others (like malls and cities), what is the better approach for improving neuroplasticity mechanisms?

## 5. Conclusion

In the last decade, VR technologies have been offering numerous possibilities for the design and development of sophisticated simulations of ADL’s that are clinically interesting, especially in the assessment and rehabilitation of ABI cognitive and functional impairments. Everyday life tasks, which would be difficult if not impossible to assess or train, using traditional neuropsychological methods, are now enabled by the use of VR technologies.

In this review, we have found that kitchens, supermarkets, shopping malls, streets, and cities are the most used scenarios for ecologically valid simulations. These are mostly presented in computer screens, HMD’s and laptops, and patients interact with them mostly via mouse, keyboard, joystick, GestureTek and Kinect.

From case studies to RCT’s, numerous studies have been carried out to clinically validate these VR-based assessment and rehabilitation environments. However, evidence stills modest and further research with bigger samples is needed. Also, a major uniformity of the traditional neuropsychological tests that are used would strengthen conclusions between different studies. With empirical studies showing greater effectiveness, these ecologically valid VR-based technologies could be of significant value for better measuring and treating ABI impairments.

### 5.1. Limitations

Based on the studies reviewed, ecologically valid VR-based simulations of ADLs offer promising advantages as ABI assessment and rehabilitation. However, it is important to refer some limitations that can affect the quality and generalizability of the findings:

1. Determining which studies to include in a literature review involves subjective decisions. Although inclusion and exclusion criteria were defined, researchers might apply them differently. In our particular case, deciding whether a VR ADLs simulation was ecologically valid involved some subjectivity. As such, there were discussion meetings among all authors to make a consensual decision.
2. Concerning the comparison of results across studies, the variability in the number and frequency of sessions and the panoply of outcome measures that were used make it difficult to analyze and generalize the results.
3. The studies reviewed aimed to include only individuals with ABI, however some studies included different diagnoses, such as MCI (Vourvopoulos et al., 2014) and MS (Krch et al., 2013), which are degenerative and might have affected the results.

### 5.2. Future work

As stated in the “*Implications for Research*” section, in order to perform a meta-analysis about the efficacy of these tools, it would be important to have guidelines about the number and frequency of sessions (for the intervention studies) and outcome measures to be used in future studies.

Additionally, the field of VR tools for assessment and rehabilitation is continually evolving, with new studies published regularly. The current review only includes articles until the end of 2021. The authors have faced a challenge in analyzing the studies and writing the manuscript while keeping the literature review up to date. It would be important to follow-up on this topic in a future review.

## Data availability statement

The raw data supporting the conclusions of this article will be made available by the authors, without undue reservation.

## Author contributions

RL proposed the review topic. RL, MSC, and SBB drafted the protocol. ALF and JL performed literature search and conducted the data extraction and analysis. ALF and JL wrote the manuscript and MSC, SBB, and RL reviewed it. All authors approved the manuscript submission.

## Funding

This work is supported by Fundação para a Ciência e Tecnologia through NOVA LINCS (UIDB/04516/2020); MACbioIDi2: Promoting the cohesion of Macaronesian regions through a common ICT platform for biomedical R & D & i (INTERREG program MAC2/1.1b/352); Ministerio de Ciencia y Educación of Spain (RTC2019-006933-7); and Conselleria d’Innovació, Universitats, Ciència i Societat Digital of the Generalitat Valenciana (CIDEXG/2022/15).

## Conflict of interest

The authors declare that the research was conducted in the absence of any commercial or financial relationships that could be construed as a potential conflict of interest.

## Publisher’s note

All claims expressed in this article are solely those of the authors and do not necessarily represent those of their affiliated organizations, or those of the publisher, the editors and the reviewers. Any product that may be evaluated in this article, or claim that may be made by its manufacturer, is not guaranteed or endorsed by the publisher.
